# Immunoglobulin-driven Complement Activation Regulates Proinflammatory Remodeling in Pulmonary Hypertension

**DOI:** 10.1164/rccm.201903-0591OC

**Published:** 2020-01-15

**Authors:** Maria G. Frid, B. Alexandre McKeon, Joshua M. Thurman, Bradley A. Maron, Min Li, Hui Zhang, Sushil Kumar, Timothy Sullivan, Jennifer Laskowsky, Mehdi A. Fini, Samantha Hu, Rubin M. Tuder, Aneta Gandjeva, Martin R. Wilkins, Christopher J. Rhodes, Pavandeep Ghataorhe, Jane A. Leopold, Rui-Sheng Wang, V. Michael Holers, Kurt R. Stenmark

**Affiliations:** ^1^ Division of Critical Care Medicine and Cardiovascular Pulmonary Research, Departments of Pediatrics and Medicine; ^2^ Division of Renal Medicine, Department of Medicine; ^4^ Division of Pulmonary and Critical Care Medicine, Department of Medicine, and; ^7^ Division of Rheumatology, Department of Medicine, School of Medicine, University of Colorado Anschutz Medical Campus, Aurora, Colorado; ^3^ Division of Cardiovascular Medicine, Department of Medicine, School of Medicine, Brigham and Women’s Hospital and Harvard Medical School, Boston, Massachusetts; ^5^ Department of Medicine and National Heart and Lung Institute, Imperial College London, London, United Kingdom; and; ^6^ Channing Division of Network Medicine, Department of Medicine, School of Medicine, Brigham Health Brigham and Women’s Hospital, Boston, Massachusetts

**Keywords:** hypoxia, inflammation, vascular remodeling, GM-CSF, biomarkers

## Abstract

**Rationale:** Pulmonary hypertension (PH) is a life-threatening cardiopulmonary disorder in which inflammation and immunity have emerged as critical early pathogenic elements. Although proinflammatory processes in PH and pulmonary arterial hypertension (PAH) are the focus of extensive investigation, the initiating mechanisms remain elusive.

**Objectives:** We tested whether activation of the complement cascade is critical in regulating proinflammatory and pro-proliferative processes in the initiation of experimental hypoxic PH and can serve as a prognostic biomarker of outcome in human PAH.

**Methods:** We used immunostaining of lung tissues from experimental PH models and patients with PAH, analyses of genetic murine models lacking specific complement components or circulating immunoglobulins, cultured human pulmonary adventitial fibroblasts, and network medicine analysis of a biomarker risk panel from plasma of patients with PAH.

**Measurements and Main Results:** Pulmonary perivascular-specific activation of the complement cascade was identified as a consistent critical determinant of PH and PAH in experimental animal models and humans. In experimental hypoxic PH, proinflammatory and pro-proliferative responses were dependent on complement (alternative pathway and component 5), and immunoglobulins, particularly IgG, were critical for activation of the complement cascade. We identified Csf2/GM-CSF as a primary complement-dependent inflammatory mediator. Furthermore, using network medicine analysis of a biomarker risk panel from plasma of patients with PAH, we demonstrated that complement signaling can serve as a prognostic factor for clinical outcome in PAH.

**Conclusions:** This study establishes immunoglobulin-driven dysregulated complement activation as a critical pathobiological mechanism regulating proinflammatory and pro-proliferative processes in the initiation of experimental hypoxic PH and demonstrates complement signaling as a critical determinant of clinical outcome in PAH.

**At a Glance Commentary**

**Scientific Knowledge on the Subject**

The preponderance of data associate complement with pulmonary arterial hypertension (PAH) via analysis of circulating complement components without studying the lung directly. In this study, we attempted to resolve this by analyzing both the local lung-specific processes in experimental animal pulmonary hypertension models and human lung specimens and the correlation of dysregulated complement to clinical outcome in patients with PAH.

**What This Study Adds to the Field**

We believe our study demonstrates, for the first time, that the immunoglobulin-driven activation of the complement cascade, and specifically its alternative pathway, in the pulmonary vascular adventitia is a critical mechanism initiating proinflammatory responses in pulmonary hypertension and PAH.

Pulmonary hypertension (PH) is a debilitating cardiopulmonary disorder with an average life expectancy <5 years from the time of diagnosis. Inflammation and immunity have emerged as critical early pathogenic elements of pulmonary arterial hypertension (PAH) (1–3). Perivascular and adventitial accumulation of monocytes and macrophages and augmented expression of proinflammatory cytokines and chemokines (GM-CSF, CCL2, CX3CL1, CXCL12, and IL6), have been consistently reported in patients with PAH and experimental preclinical PH models (4–8). Moreover, immune dysregulation has been suggested to underlie certain forms of PAH (2, 9). Nevertheless, the mechanisms triggering initial activation of the immune system and inflammation in noninfectious forms of PAH remain elusive.

The complement system is an essential component of innate immunity; however, it exerts functions beyond those of targeting pathogenic threats. Importantly, this versatile pathway of immune defense can be triggered to become a potent mediator driving various inflammatory diseases (10). Little is known about the role of complement in PH and PAH pathogenesis, particularly its regulation in hypoxic signaling—“sterile inflammation” (11)—or whether complement is important in PAH clinically. Although several studies of PAH have focused on complement activation in the circulation (12, 13), emerging evidence suggests an important role for local, tissue- or cell-specific production of complement components and activation of the complement cascade (14). Complement is activated through three major interconnected pathways: classical, alternative, and lectin (10, 15). The alternative pathway has received particular attention in diseases characterized by sterile inflammation (11, 16). A traditional view that activation of the complement cascade by antigen–antibody immune complexes involves only the classical pathway has been recently challenged by studies demonstrating that the lectin and alternative pathways can be triggered by antibodies or immune complexes (17–21). An important role of alternative pathway–driven antibody-mediated complement activation has been demonstrated in the pathophysiology of aortic aneurysm, rheumatoid arthritis, and ischemia/reperfusion (14, 16, 18–22). Importantly, even when complement activation is initiated through classical or lectin pathways, 90% of downstream complement degradation fragments can be generated through the alternative pathway amplification loop (23).

In this study, we tested the hypothesis that activation of the complement cascade, specifically involving the alternative pathway, is a critical pathobiological mechanism regulating the early proinflammatory and pro-proliferative processes that characterize experimental hypoxic PH. Furthermore, we sought to determine whether there was evidence of complement activation in rodent models of more severe PH (sugen-hypoxia, monocrotaline) and in late-stage human PAH. Finally, we elucidated whether circulating complement components can serve as biomarkers of disease outcome in human PAH.

Some of the results of these studies have been previously reported in the form of abstracts (24, 25).

## Methods

### Animal Models

Mice (male) were purchased from Jackson Laboratories: C57BL/6J, B10.D2-*Hc*^*0*^ (C5 [complement component 5]-deficient [C5^−/−^]), C3 (complement component 3)-deficient (C3^−/−^), and B6.129S2-Ighm^tm1Cgn^/J (μMT^−^ mice lacking mature B lymphocytes and thus lacking all circulating immunoglobulins) (26). Cfb (complement factor B)-deficient (Cfb^−/−^) mice were bred in-house (27). Wistar-Kyoto male rats were from Charles Rivers Laboratories. On delivery from the vendor, all animals were acclimatized for at least a week in a sea-level (SL) chamber (barometric pressure [P_B_] = 760 mm Hg) because P_B_ is 640 mm Hg at Denver altitude. In-house–bred Cfb^−/−^ mice were placed into SL chambers on weaning. Thereafter, control groups remained in SL chambers, whereas experimental groups were placed for 3 days into hypobaric (P_B_ = 380 mm Hg) hypoxic chambers (with oxygen levels approximately 12%; sample size for each SL or hypoxic group was 6–8 rats or 8–12 mice) (4, 28, 29). Six IgG-injected hypoxic μMT^−^ mice were used. Standard veterinary care was provided in compliance with institutional animal care and use committee–approved protocols at the University Colorado Denver. Specimens of bovine lung tissues were obtained from Holstein neonatal (15-d-old) male calves; the experimental hypoxic group (*n* = 7) was exposed from Day 1 after birth for 2 weeks to hypobaric hypoxia (P_B_ = 445 mm Hg), whereas age-matched controls (*n* = 6) were kept at ambient altitude (P_B_ = 640 mm Hg), as described previously (4).

Additional experimental animal information can be found in the online supplement (ANIMAL MODELS).

### Human Tissues

Human lung specimens from normal (rejected) donors and patients with idiopathic PAH (IPAH; *n* = 6, each group, Table E3 in the online supplement) were provided by the Pulmonary Hypertension Breakthrough Initiative, funded through an NHLBI R24 grant (No. R24HL123767) and the Cardiovascular Medical Research Education Fund.

Immunofluorescent and immunohistochemistry (IHC) staining and quantification, qRT-PCR, *in vitro* experiments, GM-CSF ELISA, RNAscope *in situ* hybridization, IgG injections of μMT^−^ mice, and right ventricular systolic pressure (RVSP) assessment were performed as described in the online supplement.

### Statistical Analysis

Data are presented as mean ± SEM. GraphPad Prism 6.0 (GraphPad Software Inc) was used to determine significance. Unpaired, two-tailed Student *t* test was used to compare two groups. One-way ANOVA and Sidak correction for multiple comparisons were used to compare more than two groups. The Kolmogorov-Smirnov, Shapiro-Wilk, and D’Agostino tests were used to assess for normality before applying parametric statistical tests. *P* value significance was set at 0.05.

### Developing the Complement–PAH Network

#### Patient cohorts

Patients with IPAH or heritable PAH (*n* = 218) were recruited at the National Pulmonary Hypertension service at Hammersmith Hospital, London, United Kingdom. The diagnostic criteria for IPAH or heritable PAH over the course of this study were stable: raised mean pulmonary artery pressure of more than 25 mm Hg, with pulmonary capillary wedge pressure less than 15 mm Hg (and pulmonary vascular resistance >3 Wood units) at rest with exclusion of known associated diseases. The guidelines quoted were internationally agreed. All samples and data were obtained with informed consent and local research ethics committee approval. We assessed patients for eligibility between October 24, 2002, and August 13, 2013. Patients were censored on May 15, 2014, using National Health Service records. Median follow-up was 2.2 years. At the end of the follow-up period, 55 patients had died; no patients were lost to follow-up. The mortality of this cohort was previously presented (12). Nine patients underwent lung or heart and lung transplantation and were censored at this date. Patients were not fasting and were sampled at their routine clinical appointment visits. Peripheral venous blood samples were collected using EDTA. Vacutainer tubes (BD Biosciences) were immediately put on ice, centrifuged (1,300 × *g*, 15 min) within 30 minutes of collection, and plasma aliquots were stored at −80°C until required.

#### Complement–PAH network development

Forty prognostic plasma proteins identified in a published proteomic analysis of patients with IPAH or heritable PAH (12) were evaluated, using the (incomplete) consolidated human interactome, which contains information on 170,303 protein–protein interactions (30) (Table E4). Nodes in the consolidated human interactome represent proteins, and edges represent functional associations rather than physical protein–protein binding (31). Expression data from functionally associated proteins in the complement–PAH network were used to determine whether PAH subgroups could be identified from the original PAH study population (12). We used the “elbow” method to determine the best number of clusters *K*. The elbow method ran *K*-means clustering on the data set for a range of values for *K* (e.g., 1–8) and, for each value of *K*, calculated the sum of squared errors (SSE). The optimal number is the elbow position. The underlying assumption with this method is that increasing the number of clusters beyond the elbow position will not further reduce SSE substantially. Using the elbow chart, the greatest decrease in slope for SSE across sequentially clusters was at *K* 2–3 from *K* 1–2. Therefore, we selected *K* = 2 and then used *K*-means clustering algorithm to cluster the data into two clusters. The network-based patient cluster assignment was tested for association with all-cause mortality from time of sampling by Kaplan–Meier analysis, as described previously (12). Kaplan–Meier plots illustrating events (deaths) in relation to biomarker levels were assessed by the log-rank test.

## Results

### Hypoxia Activates the Alternative Pathway of Complement

Little is known about the mechanisms involved in the early initiating stages of PH, especially those shaping pulmonary vascular microenvironments that would further perpetuate vascular remodeling. We sought to examine whether the early responses to sterile environmental stimuli (in the absence of infection or extensive injury) would be associated with complement activation, specifically via the alternative pathway that has been reported in diseases characterized by sterile inflammation (11, 16). We chose to use a commonly accepted rodent model of hypoxia-induced PH in its early stage (3-d hypoxic challenge), when the cellular processes characteristic of PH pathogenesis (accumulation of monocytes and macrophages, increased cell proliferation) are observed (4, 7, 28, 29).

**Figure 1.**
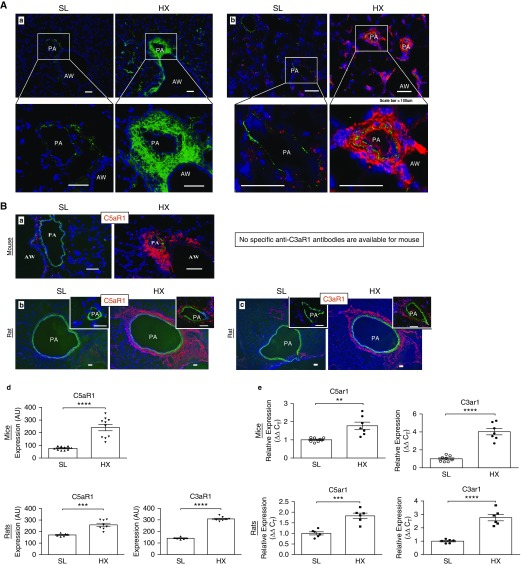
Lungs of hypoxic rodents demonstrate prominent vascular-specific deposition of complement C3 (component 3) and robust accumulation of cells expressing complement anaphylatoxin receptors. (*A*) Hypoxia-induced deposition of complement C3 in mice (*a*, green fluorochrome) and rats (*b*, red fluorochrome) is prominently observed in perivascular areas and also encompasses medial and luminal areas. (*Aa* and *Ab*) Lung cryosections were labeled with species-specific anti–complement C3 antibodies conjugated with green (*a*, for mouse) or red (*b*, for rat) fluorochrome. Cell nuclei are labeled with DAPI (blue). Scale bars, 100 μm. (*B*) Expression of receptors for complement anaphylatoxins C5a and C3a (C5aR1 and C3aR1, respectively) is markedly upregulated in the lungs of animals with experimental pulmonary hypertension. In rodents exposed to hypoxia (HX) for three days (*a*, mice; *b* and *c*, rats), cells expressing C5aR1 and C3aR1 (red fluorochrome) are markedly increased in numbers and localized to perivascular areas, whereas only a few pulmonary adventitial cells express these receptors in the lungs of sea-level (SL) animals. Lung cryosections were labeled with species-specific monoclonal antibodies against C5aR1 (mice and rats) and C3aR1 (available for rats only). Autofluorescence of elastic lamellae (green) defines tunica media, and cell nuclei are labeled with DAPI (blue). Scale bars, 100 μm. (*Bd*) Quantification of red fluorescence was performed as described in the online supplement and is presented in arbitrary units (AU). (*Be*) qRT-PCR analysis demonstrates that expression levels of C5ar1 and C3ar1 mRNA in the whole lungs of mice and rats are significantly upregulated by 3-day exposure to HX compared with SL controls. Unpaired/two-tailed test was performed for comparing two groups. **P ≤ 0.01, ***P ≤ 0.001, and ****P ≤ 0.0001. AW = airway; PA = pulmonary artery.

Lungs of 3-day hypoxic wild-type (WT) mice and rats demonstrated robust vascular-specific deposition of complement C3, whereas no C3 deposition was detected in SL animals (Figure 1A). Furthermore, markedly increased numbers of cells expressing receptors for anaphylatoxins C5a and C3a (C5aR1 and C3aR1, respectively) were observed in perivascular and periairway areas of elastic pulmonary arteries (Figures 1Ba–1Bc, quantified in Figure 1Bd, and confirmed in whole-lung lysates via RT-PCR in Figure 1Be).

**Figure 2.**
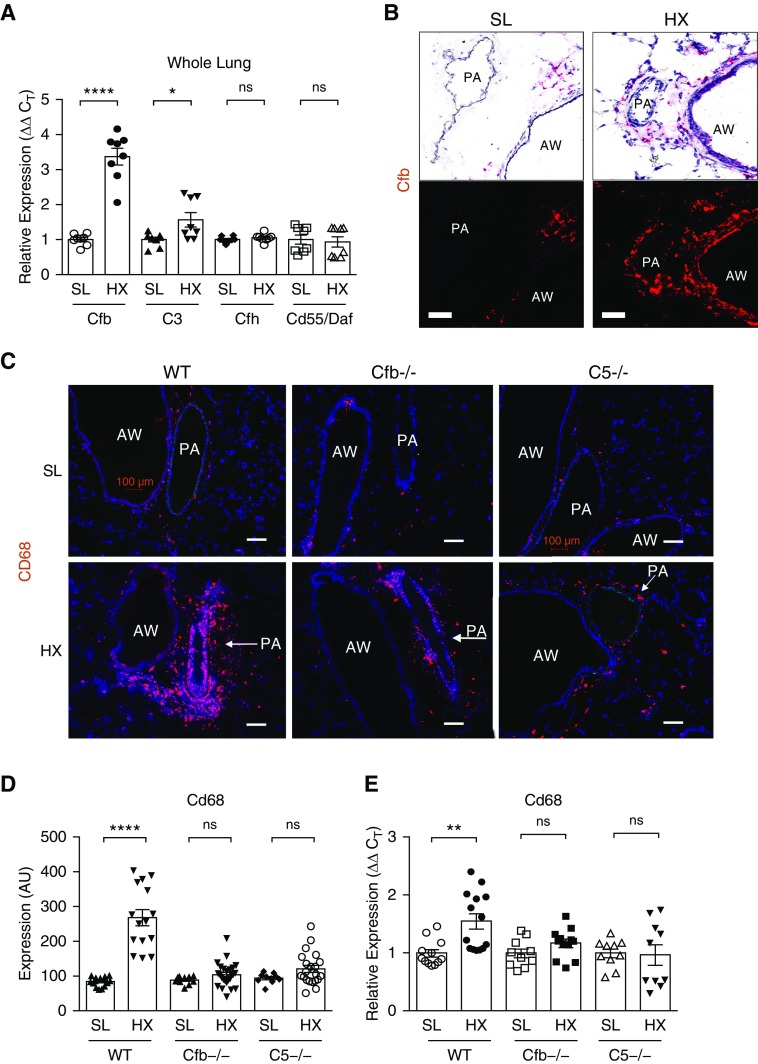
The alternative and terminal C5 (component 5) complement pathways are essential in driving hypoxia-induced proinflammatory processes in pulmonary perivascular areas. (*A* and *B*) The alternative pathway of complement is activated by hypoxic exposure. (*A*) qRT-PCR analysis of whole lung extracts from wild-type (WT) mice exposed to hypoxia (HX) for 3 days demonstrates robust augmentation of Cfb (complement factor B) and modest upregulation of complement C3 mRNA expression levels compared with sea-level (SL) controls. Unpaired/two-tailed test was performed for comparing two groups. (*B*) Lungs of 3-day HX WT mice demonstrate robust augmentation of Cfb mRNA expression, as detected via RNAscope *in situ* hybridization, in cells localized to pulmonary artery (PA) perivascular areas and airways (AWs). Fast Red chromogen (red), used for message detection in *in situ* hybridization, can be visualized by both light (upper panels) and fluorescent (bottom panels) microscopy. Gill’s hematoxylin (blue) was used for nuclear counterstaining in light microscopy imaging (upper panels). (*C–E*) Attenuated accumulation of CD68^+^ macrophages is observed in the lungs of complement (Cfb and C5)-deficient HX mice compared with WT HX counterparts, as validated by CD68 immunostaining (*C*, red fluorochrome) and its quantification (*D*) performed as described in the online supplement and presented in arbitrary units (AU), as well as by qRT-PCR of whole lung tissues (*E*). (Unpaired/two-tailed test was performed for comparing two groups in each cohort). Scale bars, 100 μm. *P < 0.05, **P ≤ 0.01, and ****P ≤ 0.0001; ns = not significant. Cfh = complement factor H; C_T_ = cycle threshold.

RT-PCR analysis of whole lungs of 3-day hypoxic mice demonstrated robust augmentation of the key activator of the complement alternative pathway, Cfb, and modest increases in complement C3 mRNA but no change in expression of regulators or inhibitors of the alternative pathway, Cfh (complement factor H), and Cd55/Daf (Figure 2A). Moreover, no hypoxia-induced changes were detected for complement C4b mRNA (defining the classical and lectin pathways; Figure E1). These findings in whole mouse lungs were corroborated by RNA-sequencing analysis of flow-sorted murine lung interstitial macrophages (IMs) in our previously published article (29), in which hypoxic IMs showed 3.2-fold increases in Cfb and concurrent decreases in Cfh (−1.43-fold) and Cd55/Daf (−2.47-fold) over SL controls (Table E1). Because these results suggested hypoxia-induced activation or amplification of the alternative pathway locally within the lung, we sought to identify the cellular source of Cfb. RNAscope *in situ* hybridization demonstrated minimal Cfb expression in SL mice, whereas robust Cfb upregulation was observed in pulmonary adventitia and airways of hypoxic mice (Figures 2B and E2). Thus, the alternative pathway and its activator Cfb emerged as critical hypoxia-induced constituents in the lung vasculature.

### Hypoxia-induced Perivascular Inflammation Is Complement Dependent

A distinctive feature of PH is early and persistent perivascular inflammation (3, 4, 32), as confirmed in 3-day hypoxic WT mice (Figure E3). Using mice genetically deficient in specific complement components, we tested the hypothesis that hypoxia-induced lung inflammation is complement dependent. Surprisingly, and in contrast to a previous publication (33), complement C3^−/−^ mice demonstrated robust accumulation of CD68^+^ macrophages, augmented Il6 and Ccl2 expression, and cell proliferation (Figure E4), potentially due to previously described generation of anaphylatoxin C5a in C3^−/−^ mice (15). Consequently, C3^−/−^ mice were not further analyzed in this study. We next tested the Cfb^−/−^ strain (to determine the role of the alternative pathway), and the C5^−/−^ strain (to define the role of the terminal C5 pathway). Although hypoxic WT mice displayed increases in perivascular CD68^+^ macrophages, the lungs of hypoxic Cfb^−/−^ and C5^−/−^ mice demonstrated decreased accumulation of CD68^+^ macrophages (Figures 2C–2E).

**Figure 3.**
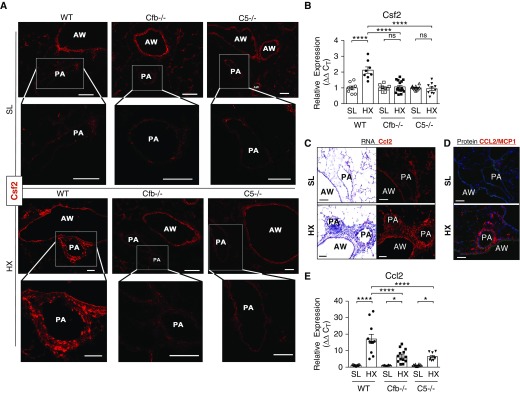
Hypoxia-induced Csf2 and Ccl2 mRNA expression is markedly augmented in the lungs of wild-type (WT) mice but is abrogated (for Csf2) or partially decreased (for Ccl2) in the lungs of complement (Cfb [complement factor B] and C5 [component 5])-deficient mice. (*A*) RNAscope *in situ* hybridization demonstrates that Csf2 expression is markedly augmented in the pulmonary arteries (PAs) of WT mice exposed to 3-day hypoxia (HX) but is abrogated in the PAs of complement (Cfb and C5)-deficient mice. Airways (AWs) of all mouse strains, both sea level (SL) and HX, maintain Csf2 expression without any visually significant change. (*B*) qRT-PCR analysis of whole murine lung tissues demonstrates that hypoxia-induced upregulation of Csf2 mRNA expression, detected in WT mice, is abrogated in complement (Cfb and C5)-deficient mice. One-way ANOVA with a Sidak multiple-comparison test with single pooled variance was performed for multiple group comparison. (*C*–*E*) HX-induced Ccl2 expression is complement (alternative and C5 pathways) dependent: levels of Ccl2 mRNA expression (*C* and *E*) and CCL2 protein expression (*D*) are markedly augmented in the lungs of 3-day HX WT mice; in contrast, the lungs of complement (Cfb and C5)-deficient HX mice demonstrate significantly decreased mRNA expression levels (*E*). One-way ANOVA with a Sidak multiple-comparison test with a single pooled variance was performed for multiple-group comparison. Scale bars, 100 μm. *P ≤ 0.05 and ****P ≤ 0.0001; ns = not significant. C_T_ = cycle threshold.

Next we examined expression and localization of Csf2/GM-CSF, a potent cytokine implicated in mobilization and proinflammatory activation of monocytes and macrophages in PAH (34). *In situ* hybridization demonstrated that, in SL-WT mice, Csf2 was localized mainly to airways but not to pulmonary vasculature, whereas robust upregulation of Csf2 expression was detected in pulmonary arteries of hypoxic WT mice (Figure 3A). Remarkably, Cfb^−/−^ and C5^−/−^ mice demonstrated abrogation of hypoxia-induced Csf2 upregulation in lung vasculature. Airways of all mouse strains, SL or hypoxic, maintained Csf2 expression without any significant visual change. This was verified by qRT-PCR demonstrating abrogation of hypoxia-induced Csf2 upregulation in mice deficient in complement (Cfb and C5; Figure 3B). In addition, we analyzed expression of a potent monocyte chemoattractant, Ccl2/MCP1. *In situ* hybridization demonstrated significant hypoxia-induced Ccl2/MCP1 upregulation in vasculature and airways (Figure 3C), whereas MCP1 protein was detected only in perivascular and adventitial areas (Figure 3D). RT-PCR analysis showed potent upregulation of Ccl2 in hypoxic WT lungs but significantly attenuated Ccl2 expression in the lungs of mice deficient in complement (Cfb and C5; Figure 3E).

### Hypoxia-induced Perivascular Cell Proliferation Is Complement Dependent

**Figure 4.**
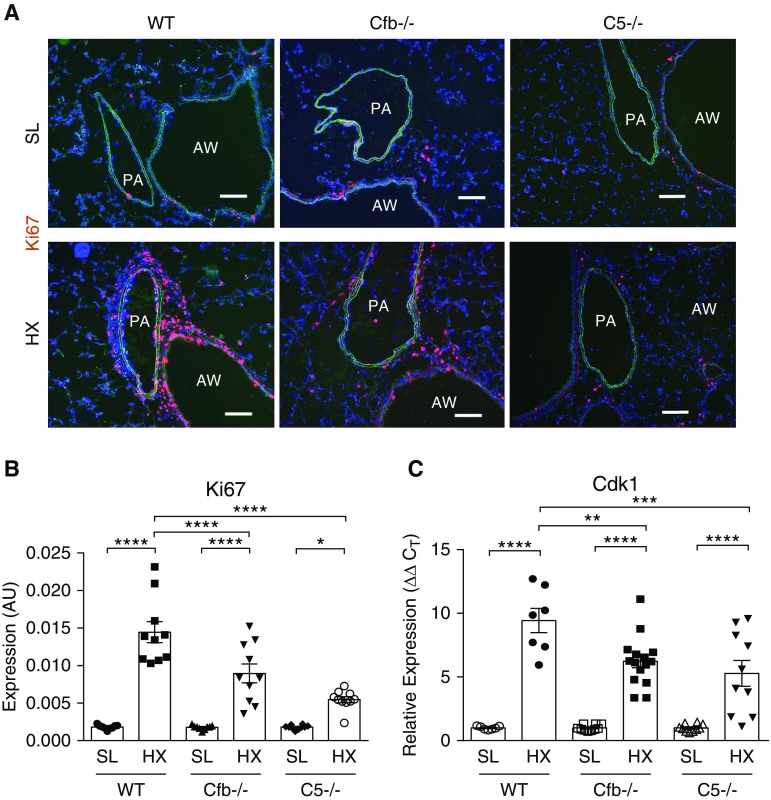
Hypoxia (HX)-induced perivascular cell proliferation is complement (alternative and C5 [component 5] pathways) dependent. (*A*) Profound increases in perivascular cell proliferative responses are observed in the lungs of wild-type (WT) mice exposed to 3-day HX, as compared with sea-level (SL) controls, but are significantly attenuated in Cfb (complement factor B)- and C5-deficient HX mice, as evaluated by immunostaining for nuclear proliferation-associated Ki67 antigen (red). Scale bars, 100 μm. (*B*) Quantification of red fluorescence was performed as described in the online supplement and is presented in arbitrary units (AU). (*C*) qRT-PCR analysis of whole lung tissues for cell cycle–progression marker Cdk1 (cyclin-dependent kinase 1) expression. One-way ANOVA with a Sidak multiple-comparison test with single pooled variance was performed for multiple-group comparison. *P < 0.05, **P ≤ 0.01, ***P ≤ 0.001, and ****P ≤ 0.0001. AW = airway; PA = pulmonary artery.

Augmented lung-cell proliferation is a distinctive characteristic of the early response to hypoxic insult (7), confirmed by increased numbers of replicating Ki67^+^ perivascular cells in hypoxic WT lungs (Figures 4A and 4B) and markedly augmented mRNA expression of Cdk1 (cyclin-dependent kinase 1) directly involved in cell-cycle progression (35) (Figure 4C). In contrast, lungs of hypoxic Cfb^−/−^ and C5^−/−^ mice displayed significantly fewer Ki67^+^cells and attenuated Cdk1 expression (Figures 4A–4C), implicating the complement signaling in partially regulating hypoxia-induced lung-cell proliferation.

Notably, RVSPs of 3-day hypoxic WT and complement-deficient mice were elevated likely because of vasoconstriction at this early time point in the disease process (Figures E5*A*–E5*C*).

### Csf2/GM-CSF Expression and Production by Cultured Pulmonary Fibroblasts Is Complement Dependent

**Figure 5.**
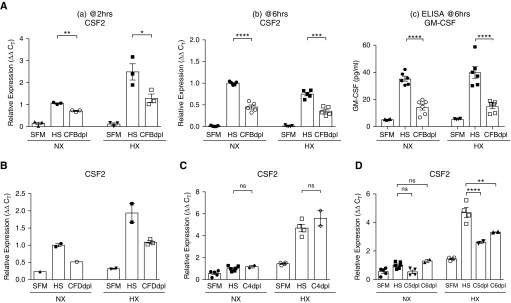
Human pulmonary adventitial fibroblasts regulate Csf2/GM-CSF expression in a complement (alternative and C5 [component 5] pathways)-dependent manner. Fibroblasts, isolated from pulmonary arteries of patients with idiopathic pulmonary arterial hypertension (PH-Fibs; *n* = 5; Table E2), were serum-starved in serum-free medium (SFM) for 72 hours and incubated with complement-sufficient normal human serum (HS) or HS depleted in specific complement components. (*A* and *B*) PH-Fibs substantially upregulate CSF2 mRNA (*Aa*, *Ab*, and *B*) and protein (*Ac*) expression in response to complement-sufficient HS, whereas their responses to serum with inhibited alternative pathway (CFBdpl [complement factor B–depleted], *Aa* and *Ab*; CFDdpl [complement factor D–depleted], *B*) are significantly attenuated. This pattern was observed under both normoxia (NX; 21% O_2_) and hypoxia (HX; 3% O_2_) conditions; however, at a 2-hour time point (three different cell populations shown, *Aa*), the levels of HX-induced CSF2 mRNA expression were higher than those in NX, which was in contrast to a 6-hour time point (*n* = 5 cell populations shown, *Ab*). The qRT-PCR findings were confirmed at a protein level via ELISA (*Ac*) using conditioned medium generated at a 6-hour time point. (*C*) Expression of CSF2 by PH-Fibs was observed to be independent of complement C4 (main component of the classical and lectin pathways). (*D*) Two-hour exposure of PH-Fibs to HS depleted in complement C5 (C5dpl) or C6 (C6dpl) resulted in substantial attenuation of CSF2 expression compared with HS under HX (3% O_2_) but not under NX (21% O_2_) conditions. *P < 0.05, **P ≤ 0.01, ***P ≤ 0.001, and ****P ≤ 0.0001; ns = not significant. C4dpl = component 4–depleted; C_T_ = cycle threshold.

Because we have previously shown that pulmonary perivascular fibroblasts from patients with PAH (termed *PH-Fibs*; Table E2) exhibit a CSF2/GM-CSF–expressing phenotype (5), we used these cells to evaluate whether the complement signaling directly regulates CSF2/GM-CSF expression and production. Exposure of serum-starved PH-Fibs to complement-sufficient human serum for 2 to 6 hours resulted in profound CSF2 mRNA increases under normoxia (21% O_2_) and, at 2 hours, under hypoxic conditions, whereas incubation in serum with a selectively inhibited alternative pathway (depleted in CFB) consistently resulted in significant attenuation of CSF2 mRNA (Figures 5Aa and 5Ab) and protein expression (Figure 5Ac). Similar findings were obtained with serum depleted of another essential activator of the alternative pathway, CFD (complement factor D; Figure 5B). On the contrary, CSF2 expression in PH-Fibs was complement C4 (classical pathway) independent (Figure 5C). Furthermore, PH-Fibs exposed to serum with inhibited terminal C5 pathway (C5-depleted sera) or with inhibited assembly of membrane-attack complex (MAC; C6-depleted sera) also demonstrated significantly decreased CSF2 expression under hypoxia (3%O_2_, 2 h) but no significant attenuation under normoxia (Figure 5D), suggesting a potential role for C5 and sublytical MAC assembly specifically in hypoxia-induced increases in CSF2 expression.

### Hypoxia-induced IgG Deposition Contributes to Complement Activation and Perivascular Proinflammatory, Pro-proliferative Processes

**Figure 6.**
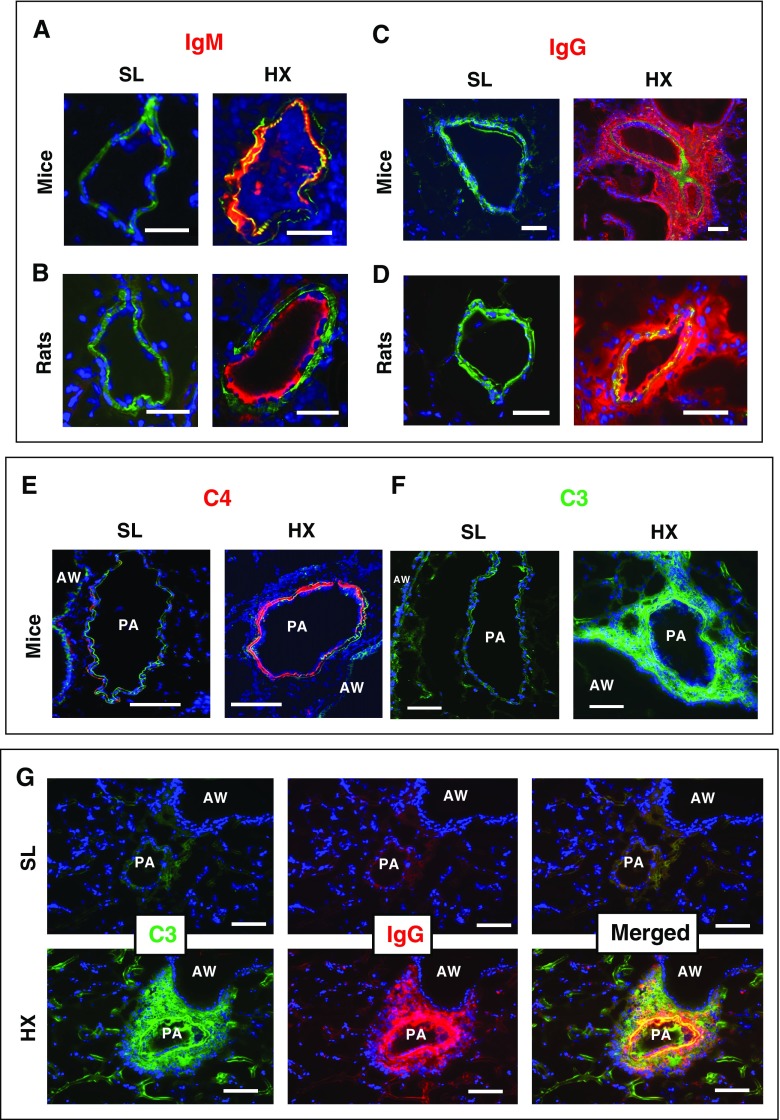
Hypoxia (HX)-induced vascular deposition patterns of IgM and IgG are highly compartmentalized and correlate with deposition patterns of complement components C4 and C3, respectively. (*A*–*D*) Highly compartmentalized patterns of IgM versus IgG deposition and complement components C4 versus C3 are observed in experimental rodents (mice and rats) exposed to 3-day HX: IgM deposition (red) in mice (*A*) and rats (*B*) is observed in the luminal and medial areas, whereas deposition of IgG (red) in mice (*C*) and rats (*D*) is detected mainly in perivascular/adventitial areas and additionally encompasses luminal/medial areas. (*E*–*G*) In 3-day HX mice, luminal/medial deposition of complement C4 (red, *E*) correlates with that of IgM (red, *A* and *B*), whereas strong perivascular (in addition to luminal/medial) deposition of complement C3 (green, *F* and *G*) clearly correlates with that of IgG (red, *C*, *D*, and *G*). No deposition of IgM, IgG, C4, or C3 was detected in the lungs of sea-level (SL) rodents. Autofluorescence of elastic lamellae (green in *A*, *B*, and *E*–*G*) or α-smooth muscle actin (green in *C* and *D*) defines tunica media. Cell nuclei are labeled with DAPI (blue). Scale bars, 100 μm. AW = airway; PA = pulmonary artery.

Having determined an essential role of complement signaling in hypoxia-induced perivascular inflammation and cell proliferation, we next sought to define its upstream triggers. Because previous studies have suggested an important role for (auto)immune mechanisms in the pathophysiology of PH (2, 9), we evaluated the contribution of immunoglobulins to hypoxia-induced complement activation and proinflammatory lung remodeling. Pulmonary vasculature of 3-day hypoxic mice and rats demonstrated profound deposition of both IgM (Figures 6A and 6B) and IgG (Figures 6C and 6D) but each in a specific compartmentalized pattern: IgM deposition was restricted to luminal/medial areas (Figures 6A and 6B), whereas IgG deposition encompassed all vascular layers but was especially prominent perivascularly (Figures 6C and 6D). Furthermore, luminal/medial IgM deposition pattern clearly correlated with that of deposited C4 (complement component of classical and lectin pathways; Figure 6E), whereas perivascular plus luminal/medial IgG deposition pattern strongly correlated with that of complement C3 (component of the alternative pathway; Figures 6F and 6G). No IgM, IgG, C4, or C3 deposition was detected in SL animals. Interestingly, although the final degradation fragments of complement components C4 and C3 (C4d and C3d, respectively) were not yet detectable at the early, 3-day hypoxic time point, they were readily observed at 3 weeks of sustained hypoxic exposure in a compartmentalized pattern that clearly correlated with the pattern of their respective native C4 and C3 components: luminal/medial for C4d and perivascular for C3d (Figure E6).

**Figure 7.**
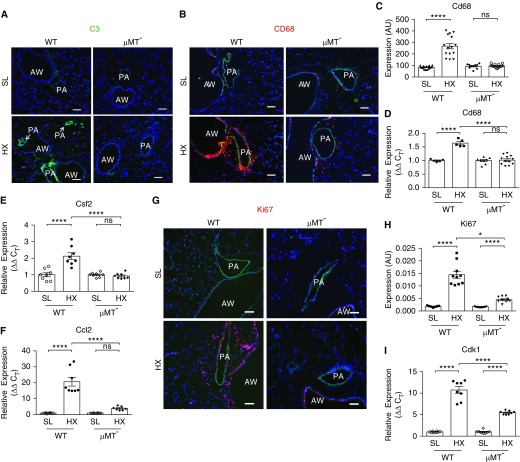
μMT mice deficient in all circulating immunoglobulins exhibit a “hypoxia-protective” phenotype. (*A*) Exposure to 3-day hypoxia (HX) fails to activate complement C3 (component 3) cascade in the lungs of μMT mice. Although the lungs of 3-day HX wild-type (WT) mice demonstrate activation of the complement cascade, as defined by strong perivascular deposition of complement C3 (green, lower left), no C3 deposition is observed in the lungs of 3-day HX μMT mice (lower right). Control sea-level (SL) mice of both strains do not display any C3 deposition. Cell nuclei are labeled with DAPI (blue). (*B*–*I*) Hypoxia-induced proinflammatory and proliferative responses are attenuated in the lungs of μMT mice. Accumulation of CD68^+^ macrophages (*B*–*D*) and Csf2 and Ccl2 cytokine/chemokine expression (*E* and *F*) are strongly (for Cd68 and Csf2) or moderately (for Ccl2) attenuated in the lungs of 3-day HX μMT^−^ mice compared with HX WT counterparts. (*B*) Immunofluorescent staining for CD68 macrophage marker (red fluorescence) and α-smooth muscle actin (green fluorescence). DAPI staining (blue) defines cell nuclei. (*C*) Quantification of red fluorescence was performed as described in the online supplement and is presented in arbitrary units (AU). (*D*–*F*) qRT-PCR analysis of whole lung tissues for mRNA expression of macrophage marker Cd68 (*D*), Csf2 (*E*), and Ccl2 (*F*). One-way ANOVA with a Sidak multiple-comparison test with single pooled variance was performed for multiple-group comparison. (*G*–*I*) Hypoxia-induced perivascular cell proliferative responses, defined by immunostaining for nuclear proliferation-associated Ki67 antigen (red, *G*, quantified in *H*) and qRT-PCR analysis of whole lung tissues for cell cycle–progression marker Cdk1 (cyclin-dependent kinase 1) expression (*I*) are significantly attenuated in the lungs of HX μMT mice compared with HX WT counterparts. (*G*) Autofluorescence of pulmonary artery elastic lamellae (green) defines tunica media; cell nuclei are labeled with DAPI (blue). *P ≤ 0.05 and ****P ≤ 0.0001; ns = not significant. Scale bars, 100 μm. AW = airway; C_T_ = cycle threshold; PA = pulmonary artery.

Having observed consistent deposition of immunoglobulins in hypoxic rodents, we proceeded with evaluating hypoxic responses in mice lacking all circulating immunoglobulins (μMT^−^ mice) (26). Although lungs of 3-day hypoxic WT mice, juxtaposed to SL-WT counterparts, demonstrated robust perivascular deposition of complement C3, profound inflammation (augmented accumulation of CD68^+^ macrophages, Cd68 and Csf2, Ccl2 mRNA expression), and increased cell proliferation (Ki67^+^ cells, Cdk1 expression; Figures 7A–7I), remarkably, μMT^−^ mice were protected from hypoxia-induced vascular changes (no detectable complement C3 deposition, reduced perivascular accumulation of CD68^+^macrophages, near-complete abrogation of Csf2, significant attenuation of Ccl2 expression, and decreases in cell proliferation; Figures 7A–7I). Interestingly, although RVSPs of 3-day hypoxic WT mice, compared with SL-WT, were augmented likely because of vasoconstriction, RVSPs of hypoxic μMT^−^ mice were almost unchanged compared with SL (Figure E7).

**Figure 8.**
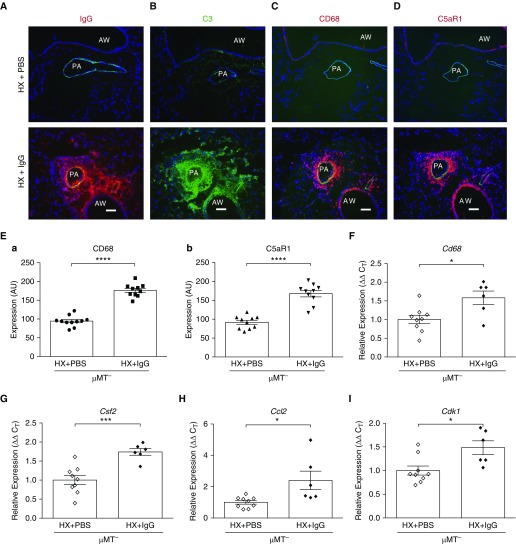
Reconstitution of circulating IgG in hypoxic μMT^−^ mice restores a proinflammatory phenotype. Five consecutive daily injections of normoxic μMT^−^ mice with normal mouse IgG (2 mg/mouse) (36), equivalent to the “loading/initiation” phase of IgG reconstitution in immune-deficient human individuals (37), was followed by 3 “blank” days and subsequently by exposure to 3-day hypobaric hypoxia (HX). (*A*) Robust deposition of IgG (red) is observed mainly in pulmonary arteries (PAs) in a perivascular-specific manner but also encompasses vascular luminal and medial compartments. Some periairway deposition is also detected. (*B*) Deposition of complement C3 (component 3; green) is prominently observed in PA perivascular areas, as well as in vascular medial and luminal compartments and partially in periairway areas. (*C* and *D*) Robust accumulation of CD68^+^and C5aR1-expressing macrophages (red) is observed in perivascular areas. Cell nuclei are labeled with DAPI (blue). Scale bars, 100 μm. (*E*) Quantification of increased accumulation of CD68^+^and C5aR1^+^cells (red fluorescence) was performed as described in the online supplement and is presented in arbitrary units (AU). Quantification of IgG and C3 deposition was not performed because the sea-level lung samples were completely negative for the staining. (*F*–*I*) qRT-PCR analysis demonstrates augmented expression of Cd68 (macrophage marker), Csf2 and Ccl2 (cytokine and chemokine, respectively), and Cdk1 (cyclin-dependent kinase 1; cell cycle–progression marker) in the whole lungs of HX immunoglobulin-deficient μMT mice that were reconstituted with circulating IgG (HX + IgG) compared with PBS-injected HX μMT mice.*P < 0.05, ***P ≤ 0.001, and ****P ≤ 0.0001. AW = airway; C5aR1 = receptor for anaphylatoxin C5a; C_T_ = cycle threshold.

Because of consistently observed perivascular colocalization of IgG, C3, and CD68^+^, C5aR1^+^, and C3aR^+^ cells, we sought to determine whether hypoxic μMT^−^ mice reconstituted with IgG to its normal circulating levels (36) using an injection protocol similar to that in human immunodeficient patients (37) (online supplement) would recapitulate the WT hypoxic phenotype. Strikingly, most features of the pathologic hypoxia-induced WT phenotype were restored (perivascular deposition of IgG and C3, augmented accumulation of CD68^+^, C5aR1^+^macrophages, increased expression of Csf2, Ccl2, and Cdk1; Figures 8A–8I). These data indicated an essential contribution of IgG to proinflammatory changes in the lung vasculature.

### Complement Cascade Is Activated in a Perivascular-Specific Manner in Various Experimental PH Models and Human PAH

**Figure 9.**
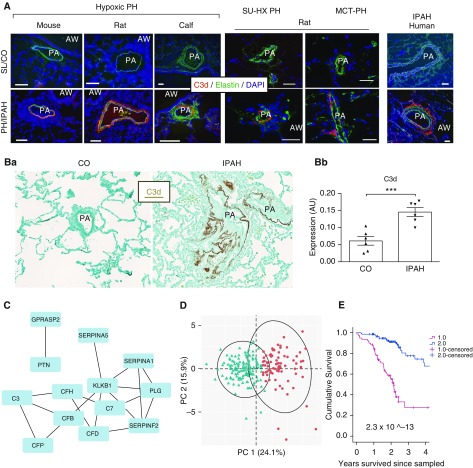
The complement cascade is activated in the lungs of experimental animal models of pulmonary hypertension (PH) and patients with idiopathic pulmonary arterial hypertension (IPAH). (*A* and *B*) Activation of the complement cascade, as defined by deposition of C3d fragment (terminal activation product of complement component 3 [C3]) is most prominently observed in a perivascular-specific manner in the lungs of experimental animal models of hypoxic PH (mouse, rat, and calf), sugen-hypoxia (SU-HX) PH and monocrotaline‐PH (MCT‐PH) rat models, and humans with IPAH. OCT-embedded lung cryosections of experimental animal specimens (*A*) and formalin-fixed paraffin-embedded sections of human lung specimens (*B*) were labeled with a biotinylated C3d-specific monoclonal antibody (mAb; red in *A*, brown in *B*), developed by Thurman and colleagues (38). This mAb distinguishes tissue-bound C3d from the intact C3 or C3b, allowing assessment of tissue-specific activation of the complement cascade. (*A*) Exposure of experimental animals to sustained hypoxia was performed for 3 weeks for mice and rats (*n* = 5, each group) and 2 weeks for calves (*n* = 5, each group); control (CO) animals were maintained at sea-level (SL) or ambient (Denver, Colorado) altitude. An image of the SU-HX rat model is shown at 2 weeks of hypoxic exposure (the most proinflammatory time point), and an image of the MCT-PH rat lung sample is shown at a 2-week time point. Autofluorescence of pulmonary artery (PA) elastic lamellae (green) defines tunica media in hypoxic experimental animal models and in human specimens, whereas α-smooth muscle actin defines tunica media in MCT-PH and SU-HX lung specimens. Images of C3d immunofluorescent staining for all analyzed human specimens are shown in Figure E8A. IPAH and normal (rejected for lung transplant) donor cohorts are described in Table E3A. Cell nuclei are labeled with DAPI (blue). Scale bars, 100 μm. (*Ba*) Immunohistochemistry (IHC) staining demonstrates C3d deposition in a medium-sized PA of a patient with IPAH. The C3d IHC signal is largely restricted to perivascular areas. Cohorts of patients with IPAH and CO (rejected for lung transplant) donors (*n* = 6, each) are described in Table E3. Images of C3d IHC staining of all analyzed human specimens (*n* = 6, each cohort) are shown in Figure E8B. (*Bb*) Quantification of IHC staining was performed via MetaMorph software, in which the dynamic range for gray intensity levels ranges between 0 (white) and 256 (black). Background levels of gray intensity are thus determined largely by the counterstain for the IHC (methyl green) and potentially represent baseline of nonspecific IHC signal to the detection system. The quantification of the IHC signal (presented in arbitrary units [AU]) for C3d revealed a 2.382-fold increase in IPAH lungs over CO donor lungs. (*C*–*E*) Plasma complement is a critical determinant of clinical outcomes in patients with PAH. (*C*) Previously published data (12) identifying circulating proteins (Table E4) with prognostic importance to patients with PAH (*n* = 218) were analyzed using a network medicine approach. Differentially expressed proteins were mapped to the consolidated human interactome resulting in a network that was enriched with complement pathway intermediaries, referred to in this article as the *complement–PAH network* (13 proteins and 18 protein–protein interactions). (*D*) Two distinct PAH patient clusters were identified based on biological information derived solely from the complement–PAH network. Oval represents the estimated cluster boundaries determined by the patient data in each cluster. (*E*) Previously published data (12) identifying circulating proteins (Table E4) with prognostic importance to patients with PAH (*n* = 218) were analyzed using a network medicine approach. A total of 37 differentially expressed proteins were mapped to the consolidated human interactome resulting in the complement–PAH network. Kaplan–Meier survival estimates in patients with PAH divided into two clusters (as shown in *D*) based on the plasma levels of proteins in the complement–PAH network are presented; thin vertical marks indicate where patients were censored during the time course. ***P ≤ 0.001. AW = airway; OCT = optimal cutting temperature compound; PC = principal component.

To test whether complement activation is sustained at later time points of hypoxic exposure and in other experimental PH models, as well as in human PAH, we evaluated deposition of C3d (the final activation/degradation fragment of complement C3), which was not detectable at an early, 3-day hypoxic exposure time point but constitutes a commonly accepted marker of complement cascade activation (38). C3d deposition was consistently observed in a perivascular-specific pattern in the lungs of experimental mice, in rats and calves with chronic (2–3 wk) hypoxic PH, and in rats with sugen-hypoxia PH and monocrotaline PH (Figures 9A and E8A). In lungs of human patients with IPAH, C3d was detected immunohistochemically in pulmonary perivascular areas, with evidence of less prominent expression within the neointima of arteries with intima thickening (Figures 9Ba and E8B). The IHC signal of C3d deposition in normal (rejected) lung donors was minimal to absent.

These data decisively demonstrated that complement activation is a longitudinal marker of PH and PAH in different experimental PH models, across species, and at different stages of the disease process. Furthermore, complement activation is initiated and maintained in the pulmonary perivascular areas.

### Plasma Complement Is a Critical Determinant of Clinical Outcomes in Patients with PAH

Next we tested whether complement signaling can also serve as a biomarker in the circulation of patients with PAH. Using unbiased analytic methods, we first aimed to determine which complement signaling proteins are clinically relevant to PAH. A biomarker panel of prognostic proteins in PAH (12) (Table E4) was mapped to the consolidated human interactome (30), which includes information on functional associations between proteins that is based on protein–protein interactions. From this approach, we observed that 13 PAH proteins were connected to at least one other PAH protein, corresponding to 18 protein–protein interactions. This network was heavily populated by complement intermediaries, particularly those of the alternative pathway (C3, CFB, CFD, CFH, and CFP) and C7 of the terminal MAC pathway. As such, this was termed the “complement–PAH network” (Figure 9C). Next we hypothesized that focusing on functionally related proteins could be informative for determining differences in outcome among patients with PAH. Therefore, a *K*-means clustering algorithm was performed on the original cohort of patients with PAH (12) using the expression profile of only proteins in the complement–PAH network (39). The elbow method was used to determine the optimal number of patient clusters (*n* = 2). The summarized expression profile of proteins in the complement–PAH network alone was sufficient to identify two distinct subgroups of patients with PAH (Figure 9D) that corresponded to significant and meaningful differences in the rate of all-cause mortality (Figure 9E).

## Discussion

This study establishes *1*) essential roles for the alternative and C5 complement pathways in initiating lung inflammation through generation of Csf2/GM-CSF and Ccl2/MCP1, perivascular monocyte and macrophage accumulation, and augmentation of cell proliferation; *2*) the critical contribution of immunoglobulins, specifically IgG, to complement-driven lung inflammation; *3*) perpetuation of perivascular-specific activation of the complement cascade in the lungs of various experimental animal PH models and humans with PAH; and *4*) complement signaling (the alternative pathway, in particular) as an important correlative determinant of clinical outcomes in patients with PAH.

The complement system plays an essential role in defensive immune processes. However, dysregulated complement activation may turn this beneficial protective system into a destructive tissue-damaging villain, as shown in various inflammatory, autoimmune, and ischemic disorders (10). Mounting experimental data unveil the relative involvement of each of the three activation pathways (classical, lectin, and alternative) in complement-mediated tissue injury and remodeling. In the present study, we identified the activated alternative pathway as an essential regulator of hypoxia-induced proinflammatory and pro-proliferative changes in the lung. Similar findings of alternative pathway activation were recently reported by high-throughput analysis of the plasma proteome in patients with PAH, in which increases in the activator of the alternative pathway, CFD, and decreases in the inhibitor CFH identified patients with PAH with high risk of mortality (12).

An important and novel finding of this study demonstrates, both *in vivo* and *in vitro*, complement (alternative and C5 pathways)-dependent vascular-specific regulation of Csf2/GM-CSF expression and production. GM-CSF has emerged as a potent key mediator of tissue inflammation (40). Locally produced at sites of inflammation, GM-CSF promotes recruitment, prosurvival reprograming, and proinflammatory activation of monocytes and macrophages and participates in induction of inflammasome (34, 41). Interestingly, among various myeloid cell types, only the inflammatory Ly6C^hi^/CCR2^+^ monocytes are vitally dependent on GM-CSF to instigate tissue inflammation and damage (41). A recent study reported increased numbers of inflammatory Ly6C^hi^/CCR2^+^ monocytes in blood and lungs of hypoxic mice (42). Consequently, it is plausible that the hypoxia-induced Ly6C^hi^/CCR2^+^ monocyte expansion is dependent on GM-CSF signaling. An elegant recent study by the Rabinovitch group demonstrated that GM-CSF is a critical mediator of monocyte and macrophage recruitment and PH and PAH development (34). Our findings corroborate this exciting report by showing that attenuated Csf2 expression corresponds with reduced monocyte and macrophage recruitment; however, the novelty of our findings is in delineating the molecular mechanisms underlying Csf2/GM-CSF regulation (i.e., complement dependence) and demonstrating that GM-CSF is an essential downstream mediator of complement-induced injury in PH. Our *in vivo* data also demonstrate complement (alternative and C5 pathways)-dependent regulation of hypoxia-induced Ccl2/MCP1 expression and cell proliferation. Several reports have suggested Csf2/GM-CSF–dependent mechanisms of Ccl2/MCP1 induction (43, 44) and cell proliferation (45). It is also plausible that complement-dependent cell-proliferative responses may be a result of terminal C5 pathway activation, which generates both pro-proliferative anaphylatoxin C5a (10) and MAC, of which the latter has been shown to induce smooth muscle cell replication at sublytical concentrations (46).

An intriguing finding of the present study is in establishing the critical contribution of immunoglobulins, specifically the IgG class, to complement activation and downstream proinflammatory changes in experimental PH and human PAH. PH and PAH have long been considered to exhibit an immune-mediated component, as demonstrated by the detection of circulating autoantibodies and activated bronchus-associated lymphoid tissue (2, 47). However, the mechanisms by which the observed autoimmune dysregulation is initiated in PH, and specifically in hypoxic forms of sterile inflammation, are unknown. Conventionally, the classical pathway is held responsible for inducing antibody-mediated complement activation, in which pentameric IgM is more effective in activating complement than monomeric IgG. However, certain IgG-immune complexes have been shown to activate the lectin pathway (20) and suggested to drive the alternative pathway (48). *In vivo*, the alternative pathway exhibits spontaneous low-grade hydrolysis of complement C3 (“tick-over” mechanism) to generate C3a and C3b fragments. C3b is short-lived but can covalently bind to certain IgG molecules immobilized on the cell surface, with the docking site within the IgG C_H_1 domain, and can form C3b_2_–IgG complexes that are more stable than the C3b itself (49). Subsequent bivalent binding of properdin results in up to 11-fold potentiation of C3 cleavage, rendering significant enhancement of alternative pathway amplification in the presence of adherent C3b–IgG complexes (48). Furthermore, tissue deposition of IgG may render this surface advantageous for alternative pathway propagation by diminishing binding of the inhibitory CFH (48). Intriguingly, the alternative pathway alone can promote *in vivo* inflammation and tissue damage and, *in vitro*, is capable of activating complement C3 (21). However, when all three pathways (classical, lectin, and alternative) are intact, the alternative pathway drives only the subsequent amplification and not the initial activation step (21). We suggest that, at least in some immune complex–mediated PH and PAH cases, the alternative pathway serves as a critical facilitator of inflammatory dysregulation in its role as a potent amplification loop following initiation by the classical or lectin pathway (as shown in our study by luminal/medial deposition of complement component C4 and supported by studies in other immune complex–mediated diseases) (20, 21, 23). However, delineation of specific activation pathways initiated by hypoxia upstream of the alternative amplification loop requires further investigation.

Our data, which show a “hypoxia-protected” phenotype of immunoglobulin-deficient μMT^−^ mice and demonstrate that IgG-reconstituted μMT mice partially restored the hypoxia-induced pathological phenotype, suggest immunoglobulins (IgG in particular) as important facilitators of the proinflammatory hypoxic phenotype. Because μMT^−^ mice were injected with normal IgG, we propose that naturally occurring, so-called “natural antibodies” (N-Abs) bind to hypoxic injury–generated neoepitopes on self-cells. N-Abs are normally present in circulation of healthy persons in the absence of exogenous antigen stimulation (“preexisting” antibodies) and exert first-response protective and regulatory functions (50). Binding of N-Abs to damaged self-cells and further activation of complement may propagate a downstream cascade of adaptive immune responses leading to generation of specific autoantibodies (51). The neoepitopes recognized by N-Abs in response to hypoxic insult are the subject of ongoing investigation by our group. In cases of more advanced PH or PAH (Figure E9) or more injurious stimuli (monocrotaline PH or elastase-induced aortic aneurism models), autoimmune reactions were suggested to be initiated or propagated by antigen-specific IgG rather than N-Abs, and specific autoantibodies have been identified (2, 20, 47). However, we speculate that in the early setting of 3-day experimental hypoxia, preexisting N-Abs are likely at play.

The preponderance of data, with the exception of a sole report (33), associate complement with PAH via analysis of circulating complement components (13, 52) without studying the lung directly. This knowledge gap casts uncertainty on the translational importance of complement to the pathogenesis of PAH. In the present study, we attempted to resolve this uncertainty by analyzing both the local lung-specific processes and the correlation of dysregulated complement to clinical outcome in patients with PAH. With regard to the latter, network medicine was used to explore integrated biological pathways that are important in PAH clinically. Our findings suggest that clinical outcomes in patients with PAH could be determined by the complement–PAH network, generating unbiased, validated, and clinically important results. This provides further context toward clarifying the molecular mechanisms underpinning the role of complement in PH and PAH. The relevance of experimental animal data to the human condition was validated by our findings of similar perivascular-specific patterns of complement activation (C3d deposition) in the lungs of PH animals and patients with IPAH. Importantly, a perivascular-specific pattern of complement activation, localization of C3ar1/C5ar1-expressing cells, and IgG deposition provides further support for the essential role of the adventitia in PH-associated inflammatory processes (7, 8).

In conclusion, the data of this study establish a causal role for complement activation and immunoglobulins in the early, initiating, proinflammatory stage of experimental hypoxic PH and propose the alternative pathway of complement signaling as a prognostic factor in clinical PAH. Dysregulated complement signaling thus emerges as a persistent longitudinal determinant of PH and PAH pathobiology. Because the etiology of PH and PAH remains elusive, further delineation of diagnostic and predictor targets in the initial asymptomatic preclinical period would facilitate development of more effective prevention and treatment strategies.

## Supplementary Material

Supplements

Author disclosures

## References

[1] Humbert M Guignabert C Bonnet S Dorfmüller P Klinger JR Nicolls MR *et al* Pathology and pathobiology of pulmonary hypertension: state of the art and research perspectives *Eur Respir J* 2019 53 1801887 30545970 10.1183/13993003.01887-2018 PMC6351340

[2] Nicolls MR Taraseviciene-Stewart L Rai PR Badesch DB Voelkel NF Autoimmunity and pulmonary hypertension: a perspective *Eur Respir J* 2005 26 1110 1118 16319344 10.1183/09031936.05.00045705

[3] Rabinovitch M Guignabert C Humbert M Nicolls MR Inflammation and immunity in the pathogenesis of pulmonary arterial hypertension *Circ Res* 2014 115 165 175 24951765 10.1161/CIRCRESAHA.113.301141 PMC4097142

[4] Frid MG Brunetti JA Burke DL Carpenter TC Davie NJ Reeves JT *et al* Hypoxia-induced pulmonary vascular remodeling requires recruitment of circulating mesenchymal precursors of a monocyte/macrophage lineage *Am J Pathol* 2006 168 659 669 16436679 10.2353/ajpath.2006.050599 PMC1606508

[5] Li M Riddle SR Frid MG El Kasmi KC McKinsey TA Sokol RJ *et al* Emergence of fibroblasts with a proinflammatory epigenetically altered phenotype in severe hypoxic pulmonary hypertension *J Immunol* 2011 187 2711 2722 21813768 10.4049/jimmunol.1100479 PMC3159707

[6] Savai R Pullamsetti SS Kolbe J Bieniek E Voswinckel R Fink L *et al* Immune and inflammatory cell involvement in the pathology of idiopathic pulmonary arterial hypertension *Am J Respir Crit Care Med* 2012 186 897 908 22955318 10.1164/rccm.201202-0335OC

[7] Stenmark KR Yeager ME El Kasmi KC Nozik-Grayck E Gerasimovskaya EV Li M *et al* The adventitia: essential regulator of vascular wall structure and function *Annu Rev Physiol* 2013 75 23 47 23216413 10.1146/annurev-physiol-030212-183802 PMC3762248

[8] Tieu BC Lee C Sun H Lejeune W Recinos A III Ju X *et al* An adventitial IL-6/MCP1 amplification loop accelerates macrophage-mediated vascular inflammation leading to aortic dissection in mice *J Clin Invest* 2009 119 3637 3651 19920349 10.1172/JCI38308 PMC2786788

[9] Park SH Chen WC Durmus N Bleck B Reibman J Riemekasten G *et al* The effects of antigen-specific IgG1 antibody for the pulmonary-hypertension-phenotype and B cells for inflammation in mice exposed to antigen and fine particles from air pollution *PLoS One* 2015 10 e0129910 26079807 10.1371/journal.pone.0129910 PMC4469456

[10] Ricklin D Reis ES Lambris JD Complement in disease: a defence system turning offensive *Nat Rev Nephrol* 2016 12 383 401 27211870 10.1038/nrneph.2016.70 PMC4974115

[11] Chen GY Nuñez G Sterile inflammation: sensing and reacting to damage *Nat Rev Immunol* 2010 10 826 837 21088683 10.1038/nri2873 PMC3114424

[12] Rhodes CJ Wharton J Ghataorhe P Watson G Girerd B Howard LS *et al* Plasma proteome analysis in patients with pulmonary arterial hypertension: an observational cohort study *Lancet Respir Med* 2017 5 717 726 28624389 10.1016/S2213-2600(17)30161-3 PMC5573768

[13] Zhang J Zhang Y Li N Liu Z Xiong C Ni X *et al* Potential diagnostic biomarkers in serum of idiopathic pulmonary arterial hypertension *Respir Med* 2009 103 1801 1806 19703762 10.1016/j.rmed.2009.07.017

[14] Holers VM Targeting mechanisms at sites of complement activation for imaging and therapy *Immunobiology* 2016 221 726 732 25979851 10.1016/j.imbio.2015.04.005 PMC4627863

[15] Huber-Lang M Sarma JV Zetoune FS Rittirsch D Neff TA McGuire SR *et al* Generation of C5a in the absence of C3: a new complement activation pathway *Nat Med* 2006 12 682 687 16715088 10.1038/nm1419

[16] Thurman JM Triggers of inflammation after renal ischemia/reperfusion *Clin Immunol* 2007 123 7 13 17064966 10.1016/j.clim.2006.09.008 PMC1888143

[17] Banda NK Wood AK Takahashi K Levitt B Rudd PM Royle L *et al* Initiation of the alternative pathway of murine complement by immune complexes is dependent on N-glycans in IgG antibodies *Arthritis Rheum* 2008 58 3081 3089 18821684 10.1002/art.23865 PMC2574875

[18] Ji H Ohmura K Mahmood U Lee DM Hofhuis FM Boackle SA *et al* Arthritis critically dependent on innate immune system players *Immunity* 2002 16 157 168 11869678 10.1016/s1074-7613(02)00275-3

[19] Zhou HF Yan H Stover CM Fernandez TM Rodriguez de Cordoba S Song WC *et al* Antibody directs properdin-dependent activation of the complement alternative pathway in a mouse model of abdominal aortic aneurysm *Proc Natl Acad Sci USA* 2012 109 E415 E422 22308431 10.1073/pnas.1119000109 PMC3289386

[20] Zhou HF Yan H Bertram P Hu Y Springer LE Thompson RW *et al* Fibrinogen-specific antibody induces abdominal aortic aneurysm in mice through complement lectin pathway activation *Proc Natl Acad Sci USA* 2013 110 E4335 E4344 24167262 10.1073/pnas.1315512110 PMC3831981

[21] Banda NK Takahashi K Wood AK Holers VM Arend WP Pathogenic complement activation in collagen antibody-induced arthritis in mice requires amplification by the alternative pathway *J Immunol* 2007 179 4101 4109 17785849 10.4049/jimmunol.179.6.4101

[22] Holers VM Demoruelle MK Kuhn KA Buckner JH Robinson WH Okamoto Y *et al* Rheumatoid arthritis and the mucosal origins hypothesis: protection turns to destruction *Nat Rev Rheumatol* 2018 14 542 557 30111803 10.1038/s41584-018-0070-0 PMC6704378

[23] Harboe M Ulvund G Vien L Fung M Mollnes TE The quantitative role of alternative pathway amplification in classical pathway induced terminal complement activation *Clin Exp Immunol* 2004 138 439 446 15544620 10.1111/j.1365-2249.2004.02627.x PMC1809239

[24] Frid M McKeon A Li M Sullivan T Zhang H Riddle S *et al* Hypoxia-induced pulmonary inflammation and remodeling are dependent on perivascular-specific complement activation via the alternative pathway [abstract] *Am J Respir Crit Care Med* 2018 197 A4622

[25] Frid MG McKeon BA Li M Sullivan T Zhang H Kumar S *et al* Hypoxia-induced pulmonary inflammation and cell proliferation are complement- and antibody-driven [abstract] *Am J Respir Crit Care Med* 2019 199 A1989

[26] Kitamura D Roes J Kühn R Rajewsky K A B cell-deficient mouse by targeted disruption of the membrane exon of the immunoglobulin mu chain gene *Nature* 1991 350 423 426 1901381 10.1038/350423a0

[27] Thurman JM Lenderink AM Royer PA Coleman KE Zhou J Lambris JD *et al* C3a is required for the production of CXC chemokines by tubular epithelial cells after renal ischemia/reperfusion *J Immunol* 2007 178 1819 1828 17237432 10.4049/jimmunol.178.3.1819

[28] Li M Riddle S Zhang H D’Alessandro A Flockton A Serkova NJ *et al* Metabolic reprogramming regulates the proliferative and inflammatory phenotype of adventitial fibroblasts in pulmonary hypertension through the transcriptional corepressor C-terminal binding protein-1 *Circulation* 2016 134 1105 1121 27562971 10.1161/CIRCULATIONAHA.116.023171 PMC5069179

[29] Pugliese SC Kumar S Janssen WJ Graham BB Frid MG Riddle SR *et al* A time- and compartment-specific activation of lung macrophages in hypoxic pulmonary hypertension *J Immunol* 2017 198 4802 4812 28500078 10.4049/jimmunol.1601692 PMC5501258

[30] Menche J Sharma A Kitsak M Ghiassian SD Vidal M Loscalzo J *et al* Disease networks: uncovering disease-disease relationships through the incomplete interactome *Science* 2015 347 1257601 25700523 10.1126/science.1257601 PMC4435741

[31] Samokhin AO Stephens T Wertheim BM Wang RS Vargas SO Yung LM *et al* NEDD9 targets *COL3A1* to promote endothelial fibrosis and pulmonary arterial hypertension *Sci Transl Med* 2018 10 eaap7294 29899023 10.1126/scitranslmed.aap7294 PMC6223025

[32] Vergadi E Chang MS Lee C Liang OD Liu X Fernandez-Gonzalez A *et al* Early macrophage recruitment and alternative activation are critical for the later development of hypoxia-induced pulmonary hypertension *Circulation* 2011 123 1986 1995 21518986 10.1161/CIRCULATIONAHA.110.978627 PMC3125055

[33] Bauer EM Zheng H Comhair S Erzurum S Billiar TR Bauer PM Complement C3 deficiency attenuates chronic hypoxia-induced pulmonary hypertension in mice *PLoS One* 2011 6 e28578 22194859 10.1371/journal.pone.0028578 PMC3237464

[34] Sawada H Saito T Nickel NP Alastalo TP Glotzbach JP Chan R *et al* Reduced BMPR2 expression induces GM-CSF translation and macrophage recruitment in humans and mice to exacerbate pulmonary hypertension *J Exp Med* 2014 211 263 280 24446489 10.1084/jem.20111741 PMC3920564

[35] Morgan DO Cell cycle: principles of control Lawrence E London New Science Press 2007 30

[36] Klein-Schneegans AS Kuntz L Fonteneau P Loor F Serum concentrations of IgM, IgG1, IgG2b, IgG3 and IgA in C57BL/6 mice and their congenics at the lpr (lymphoproliferation) locus *J Autoimmun* 1989 2 869 875 2619870 10.1016/0896-8411(89)90013-9

[37] Jolles S Orange JS Gardulf A Stein MR Shapiro R Borte M *et al* Current treatment options with immunoglobulin G for the individualization of care in patients with primary immunodeficiency disease *Clin Exp Immunol* 2015 179 146 160 25384609 10.1111/cei.12485 PMC4298393

[38] Thurman JM Kulik L Orth H Wong M Renner B Sargsyan SA *et al* Detection of complement activation using monoclonal antibodies against C3d *J Clin Invest* 2013 123 2218 2230 23619360 10.1172/JCI65861 PMC3635726

[39] Oldham WM Oliveira RKF Wang RS Opotowsky AR Rubins DM Hainer J *et al* Network analysis to risk stratify patients with exercise intolerance *Circ Res* 2018 122 864 876 29437835 10.1161/CIRCRESAHA.117.312482 PMC5924425

[40] Becher B Tugues S Greter M GM-CSF: from growth factor to central mediator of tissue inflammation *Immunity* 2016 45 963 973 27851925 10.1016/j.immuni.2016.10.026

[41] Croxford AL Lanzinger M Hartmann FJ Schreiner B Mair F Pelczar P *et al* The cytokine GM-CSF drives the inflammatory signature of CCR2+ monocytes and licenses autoimmunity *Immunity* 2015 43 502 514 26341401 10.1016/j.immuni.2015.08.010

[42] Florentin J Coppin E Vasamsetti SB Zhao J Tai YY Tang Y *et al* Inflammatory macrophage expansion in pulmonary hypertension depends upon mobilization of blood-borne monocytes *J Immunol* 2018 200 3612 3625 29632145 10.4049/jimmunol.1701287 PMC5940510

[43] Owen JL Torroella-Kouri M Handel-Fernandez ME Iragavarapu-Charyulu V GM-CSF up-regulates the expression of CCL2 by T lymphocytes in mammary tumor-bearing mice *Int J Mol Med* 2007 20 129 136 17549399

[44] Tanimoto A Murata Y Wang KY Tsutsui M Kohno K Sasaguri Y Monocyte chemoattractant protein-1 expression is enhanced by granulocyte-macrophage colony-stimulating factor via Jak2-Stat5 signaling and inhibited by atorvastatin in human monocytic U937 cells *J Biol Chem* 2008 283 4643 4651 18089573 10.1074/jbc.M708853200

[45] Zhu SN Chen M Jongstra-Bilen J Cybulsky MI GM-CSF regulates intimal cell proliferation in nascent atherosclerotic lesions *J Exp Med* 2009 206 2141 2149 19752185 10.1084/jem.20090866 PMC2757868

[46] Niculescu F Badea T Rus H Sublytic C5b-9 induces proliferation of human aortic smooth muscle cells: role of mitogen activated protein kinase and phosphatidylinositol 3-kinase *Atherosclerosis* 1999 142 47 56 9920505 10.1016/s0021-9150(98)00185-3

[47] Colvin KL Cripe PJ Ivy DD Stenmark KR Yeager ME Bronchus-associated lymphoid tissue in pulmonary hypertension produces pathologic autoantibodies *Am J Respir Crit Care Med* 2013 188 1126 1136 24093638 10.1164/rccm.201302-0403OC PMC3863738

[48] Karsten CM Köhl J The immunoglobulin, IgG Fc receptor and complement triangle in autoimmune diseases *Immunobiology* 2012 217 1067 1079 22964232 10.1016/j.imbio.2012.07.015

[49] Sahu A Pangburn MK Covalent attachment of human complement C3 to IgG: identification of the amino acid residue involved in ester linkage formation *J Biol Chem* 1994 269 28997 29002 7961863

[50] Holodick NE Rodríguez-Zhurbenko N Hernández AM Defining natural antibodies *Front Immunol* 2017 8 872 28798747 10.3389/fimmu.2017.00872 PMC5526850

[51] Holers VM Banda NK Complement in the initiation and evolution of rheumatoid arthritis *Front Immunol* 2018 9 1057 29892280 10.3389/fimmu.2018.01057 PMC5985368

[52] Zhang X Hou HT Wang J Liu XC Yang Q He GW Plasma proteomic study in pulmonary arterial hypertension associated with congenital heart diseases *Sci Rep* 2016 6 36541 27886187 10.1038/srep36541 PMC5122864

