# Supplementary material for: Immunoglobulin-driven Complement Activation Regulates Proinflammatory Remodeling in Pulmonary Hypertension
Source: Am J Respir Crit Care Med. 2020 Jan 15;201(2):224–39. doi: 10.1164/rccm.201903-0591OC (PMC6961733; doi:10.1164/rccm.201903-0591OC)
Supplement: Supplements [file rccm.201903-0591OC.html]

Immunoglobulin-driven Complement Activation Regulates Proinflammatory Remodeling in Pulmonary Hypertension | American Journal of Respiratory and Critical Care Medicine

- frid\_data\_supplement.pdf (4 MB)
- disclosures.pdf (6 MB)
