# Supplementary material for: Immunoglobulin-driven Complement Activation Regulates Proinflammatory Remodeling in Pulmonary Hypertension
Source: Am J Respir Crit Care Med. 2020 Jan 15;201(2):224–39. doi: 10.1164/rccm.201903-0591OC (PMC6961733; doi:10.1164/rccm.201903-0591OC)
Supplement: Supplements [file rccm.201903-0591OC_frid_data_supplement.pdf]

Immunoglobulin-Driven Complement Activation Regulates Pro-Inflammatory Remodeling in Pulmonary Hypertension

Maria G. Frid, B. Alexandre McKeon, Joshua M. Thurman, Bradley A. Maron, Min Li, Hui Zhang, Sushil Kumar, Timothy Sullivan, Jennifer Laskowsky, Mehdi A. Fini, Samantha Hu, Rubin M. Tuder, Aneta Gandjeva, Martin R. Wilkins, Christopher J. Rhodes, Pavandeep Ghataorhe, Jane A. Leopold, Rui-Sheng Wang, V. Michael Holers, and Kurt R. Stenmark

ONLINE DATA SUPPLEMENT

ONLINE DATA SUPPLEMENT

MATERIALS and METHODS

Animal models

In the present study, we chose to use two rodent models (mice and rats) both to expand experimental findings cross-species, and due to limited availability of antibodies for immunostaining per species. Mice deficient in complement factor B (defined as Cfb-/-), were generated and backcrossed onto C57BL/6J mice for seven generations as previously described (1). For all experiments, 10-week-old (age at euthanization) male mice were used. Wistar-Kyoto (WKY) 10-week old male rats were purchased from Charles River laboratories, (Wilmington, MA). Upon delivery, all animals were acclimatized for a week in a sea-level chamber (760 mmHg) since Denver altitude is 640 mmHg. Thereafter, control sea-level groups (n=6-8 for rats and n=8-12 for mice) remained in sea-level chambers, whereas experimental groups were placed for 3 days into hypobaric (P_B=380 mmHg) hypoxic chambers (with oxygen levels approximately 12%) (n=6-8 for rats and n=8-12 for mice). Animals were euthanized and blood/tissues collected. Standard veterinary care was used in compliance with Institutional Animal Care and Use Committee-approved protocols at University of Colorado Denver. One-day-old Holstein calves were purchased from Laluna dairy farm (Fort Collins, CO). After conclusion of experiments (2-week exposure to hypobaric hypoxia (P_B = 445 mmHg) or ambient altitude (Denver, CO; P_B = 640 mmHg), animals were euthanized by overdose of sodium pentobarbital (160 mg/kg body weight). Standard veterinary care was used following institutional guidelines at the Department of Physiology, School of Veterinary Medicine, Colorado State University (Fort Collins, CO).

wherein the experimental hypoxic group (n=7) was exposed for 2 weeks to hypobaric hypoxia (P_B=445 mmHg), while age-matched controls (n=6) were kept at ambient altitude (Denver, CO; P_B=640 mmHg) (2).

Reconstitution of circulating IgG in μMT immunoglobulin-deficient mice (n=6) was performed similar to methods published for IgG reconstitution in human immunodeficient patients (3). Briefly, 5 consecutive daily s.c. injections with normal mouse IgG (2 mg/mouse (4); Jackson ImmunoResearch Labs, West Grove, PA, cat. #015-000-002) were followed by 3 blank days and then by exposure to 3-day hypobaric hypoxia or sea-level normoxia as described above. Control hypoxic mice (n=9) were injected with PBS.

In vivo right ventricular systolic pressure (RVSP) assessment

Mice were anesthetized with isoflurane (induction for 2 minutes at 5% concentration and maintained at 2-3% for duration of assessment) mixed with room air or 100% O2 using a Surgivet Isotec 4 precision vaporizer (Smiths Medical, Minneapolis, MN). Once mice were sedated, they were placed supine while spontaneously breathing the isoflurane/air mix through a rodent nosecone. A 27-gauge needle attached to a fluid filled disposable pressure transducer (Hospira, Inc., Lake Forest, IL) was introduced percutaneously into the thorax via a subxiphoid approach. Right ventricular systolic pressure (RVSP) were verified in real time and recorded using a MP100 data acquisition system with AcqKnowledge software version 3.9.1-100M (Biopac Systems, Inc., Goleta, CA). Animals from chronic hypoxia exposure were kept in hypoxic conditions until immediately before RVSP measurement. After hemodynamic

measurements, blood was collected from the heart and mice were euthanized via bilateral thoracotomy.

Immunofluorescent (IF) and iImmunohistochemical (IHC) staining

IF staining: Freshly obtained animal tissue samples were embedded in O.C.T. (Sakura FineTek, Torrancve, CA) and frozen at -80oC. For indirect immunostaining (single- or double-labeling), tissue cryosections (5 μm) were fixed in cold methanol:acetone (1:1). Nonspecific binding was blocked with DAKO Antibody Diluent (DAKO Inc., Carpinteria, CA), and sections were incubated overnight at 4°C with the following primary antibodies (Abs): mouse biotinylated-C3d/29 mAb with a wide species cross-reactivity, that recognizes iC3b and/or C3d, but not intact C3 or C3b (5); anti-rat C3aR1 (clone 74/rC3aRZ1, LSBio, Seattle, WA); anti-rat C5aR1 (CD88, clone R63, Santa Cruz Biotechnology, Santa Cruz, CA); anti-mouse complement C4 (clone 16D2) and anti-rat complement C4d (rabbit pAb) from Hycult Biotech (Plymouth Meeting, PA); CD11b (integrin μM, clone OX42, cross-reactive with rat and mouse, Santa Cruz); rat anti-mouse mAbs against CD88/C5aR1 (clone 10/92, BioRad, Hercules, CA); CD68 (MCA-1957T, Bio-Rad Laboratories, Hercules, CA); CD45 (clone 30-F11, eBioscience Inc., San Diego, CA); MHC class II (clone ER-TR3, Santa Cruz); MAC3 (CD107b/LAMP-2, clone GL2A7, Novus Biologicals, Littleton, CO); rabbit monoclonal Abs with wide species cross-reactivity against proliferation-associated nuclear antigen Ki67 (clone SP6, Thermo Scientific, Waltham, MA); and FITC-conjugated goat F(ab)'2 fragment to mouse complement C3 (MP Biomedicals, Santa Ana, CA). For single-labeling, two different staining methods were used: (1) biotinylated secondary Abs (Vector Laboratories, Burlingame, CA) followed by Streptavidin-Alexa-594 (Invitrogen/Molecular Probes, Eugene, OR), or (2) VectaFluor Excel R.T.U. Antibody Kit

DyLight-594 (anti-mouse or anti-rabbit IgG, depending on primary Abs used; purchased from Vector Laboratories). For double-labeling secondary Abs, appropriate species-specific biotinylated Abs were used, followed by Streptavidin conjugated to either Alexa-594 (red) or Alexa-488 (green). All reagents were used at dilutions recommended by the manufacturer. Immunolabeled sections were mounted in VectaShield/DAPI mounting medium (Vector Laboratories).

IHC staining on human lung sections was performed via standard technique using reagents from Vector Laboratories, briefly: after deparaffinization, antigen retrieval (30 mins boiling in pressure cooker in citrate buffer, pH 6.0) and blocking steps (avidin, 15 mins, and biotin, 15 mins), primary biotinylated anti-C3d mouse mAbs (1:300, (1) were added for 1 hr at RT, followed by RTU horseradish peroxidase (30 mins at RT) and development in DAB (5 mins). Both fluorescent- and IHC-labeled tissue sections were scanned on Leica Aperio VERSA 8 Digital Pathology Scanner with fluorescence, and bright-field slide scanner with HC PLAN APO 20x/0.70 objective lenses with Leica Image Scope acquisition software (Leica Biosystems Imaging, Inc.)

Quantification of IHC and IF staining

For IHC: Stained sections were Aperio-scanned (see above), randomized images were extracted from the scanned files and analyzed via Metamorph software. Graphs plotted wherein "Expression" represents percent threshold and "artificial units" (AU) represent the product area (pixel) time grey intensity (6).

For IF: Tissue sections with immunofluorescent staining were scanned on Leica-Aperio Versa 8 system at 20x magnification. The acquired images were then analyzed using the Aperio

ImageScope software v12.4.2 with the Area quantification FL v1 algorithm. The red fluorescence threshold was selected to eliminate the out-of-fluorescence background. The entire lung section area was selected for quantification and the red fluorescence was normalized to the total lung area and the resulting ratio is presented as “Expression” in Artificial Units (AU).

Quantative real-time PCR

RNA for quantitative RT-PCR from lung and liver tissue was isolated using QIAzol Lysis Reagent (Qiagen, Germantown, MD) according to the manufacturer’s instructions. RNA quality and quantity were analyzed using NanoDrop, and TapeStation by the Genomics and Microarray Core Cancer Center at the University of Colorado Anschutz Medical Campus. Total RNA from cultured cells was isolated using Qiagen RNeasy Kit (Qiagen, Valencia, CA) according to the manufacturer’s instructions. First strand cDNA synthesis was done using iScript cDNA Synthesis Kit (Bio-Rad, Hercules, CA). Quantitative RT-PCR was performed using TaqMan probes (**Suppl. Table 5**) with reagents for ABI-7500 Real Time PCR System (Applied Biosystems, Grand Island, NY), according to the manufacturer’s instructions.

Gene expression fold change was calculated after normalization to Hprt1 using the D/D Ct method.

In situ hybridization

RNAscope in situ hybridization platform (probes, reagents, methodology) was used (ACDBio, Newark, CA). Briefly, frozen OCT-embedded mouse lung tissue sections (prepared as described above, sectioned at 10um thickness) were fixed in cold 10% buffered formalin, hybridized with CCL2/MCP1 or CSF2/GM-CSF antisense mouse-specific probes and processed according to the

manufacturer's instructions. The bound probes were detected using Fast Red kit and counterstained with 50% Gill's hematoxyllin. Both positive and negative control probes were run in parallel to confirm specificity of the target probes. Both fluorescent and bright-field images were acquired (since bound Fast Red can be visualized in both channels) using Zeiss microscope with AxioVision digital imaging system.

<u>*In vitro experiments*</u>

Human pulmonary fibroblasts, isolated from the adventitial layer of the main pulmonary artery of IPAH patients (n= 3, **Suppl. Table 2**), were kindly provided by Dr. N.W. Morrell (University of Cambridge, UK). Human lung fibroblasts, isolated from the distal lung lobe of IPAH patients (n= 2, **Suppl. Table 2**), were kindly provided by Dr. C. Feghali-Bostwick (Medical University of South Carolina, USA). The rationale for using PH-Fibs, and not fibroblasts obtained from normal donors (CO-Fibs), was based on our previous publication (7) where we showed that CO-Fibs exhibit negligible expression levels of CSF2/GM-CSF. Therefore, any manipulations attempting to lower expression levels of this cytokine would not have been feasible in CO-Fibs. All fibroblast populations were cultured in DMEM supplemented with 10% FBS and used for experiments between passages 5-8. Cells were plated onto 6-well plates, and upon reaching 60-70% confluence, rinsed with PBS and growth medium replaced with serum-free medium (SFM) for 72 hrs. Thereafter, medium was replaced under normoxic (NX, 21%O_2) or hypoxic (HX, 3% O_2) conditions for the specific experimental time points with: a) fresh SFM, b) 10% normal human serum (HS); and c) human serum depleted of specific complement components: complement factor B (CFBdpl), complement factor D (CFDdpl) and complement components C5 (C5dpl), C3 (C3dpl), C4 (C4dpl) and C6 (C6dpl). All complement-sufficient or complement-

depleted sera products were purchased from Complement Technology, Inc. (Tyler, TX, USA). After the specified incubation periods, conditioned medium from cells was collected, snap-frozen, and stored at -80oC for ELISA.

GM-CSF ELISA

Cell culture supernatant was collected from cells after the specified incubation period, and GM-CSF was quantified using a human GM-CSF Quantikine sandwich ELISA (R&D Systems) as per the manufacturer's protocol with a quantitative detection range of 7.8 - 500 pg/ml.

Suppl. Fig. E-1 shows, via RT-PCR analysis, that no changes in expression of C4b are detected in lung tissues of WT HX mice compared to SL controls.

Supplemental Table E-1 presents RNAseq analysis of flow-sorted lung interstitial macrophages (IMs) isolated from 4-day hypoxic mice (IM-HX) or SL control mice (IM-SL) to demonstrate increases in Cfb, and concurrent decreases in Cd55/Daf and Cfh in IM-HX vs IM-SL macrophages (8).

Suppl. Fig. E-2 displays Positive and Negative Controls for RNAscope *in situ hybridization.*

Suppl. Fig. E-3 illustrates robust hypoxia-induced (HX) perivascular accumulation of CD45+ leukocytes, most of which are CD68+, CD11b+ macrophages of a pro-inflammatory (MAC3+, MHCII+) phenotype. SL: sea-level controls.

Suppl. Fig. E-4 presents immunostaining and RT-PCR data confirming pro-inflammatory pulmonary characteristics of hypoxic complement C3-deficient (C3-/-) mice (robust perivascular

accumulation of CD68+ macrophages, augmented pro-inflammatory Ccl2 and Il6 cytokine expression, and increased cell proliferation).

Suppl. Fig. E-5 demonstrates that: (A) right ventricular systolic pressures (RVSPs) of 3-day hypoxic (HX) mice (wild-type (WT) and complement-deficient (Cfb-/- an C5-/-)) are elevated over those of sea-level (SL) controls – and (B) suggests that this is due to vasoconstriction because RVSPs of experimental (”3-day hypoxic”) mice breathing 100% oxygen during RVSP measurement are significantly attenuated. (C): Importantly, mice kept in sea-level (SL) chambers for several weeks, but taken out to Denver altitude (DA; 5,407 ft) for RVSP measurements, also demonstrate RVSPs higher that those of SL mice breathing 100% oxygen during RVSP assessment.

Supplemental Table E-2 presents clinical characteristics, demographics and pathological diagnoses of PAH patients, from whose pulmonary arteries adventitial fibroblasts were derived.

Suppl. Fig. E-6 shows compartmentalization of deposition pattern of complement fragments C4d (luminal areas, red) and C3d (perivascular areas, red) in pulmonary arteries of 3-week hypoxic WKY rats.

Suppl. Fig. E-7 demonstrates that, while RVSPs of 3-day hypoxic WT mice compared to SL-WT were augmented (likely due to vasoconstriction as suggested in E-5 above), RVSPs of 3-day hypoxic μMT- mice were almost unchanged compared to their SL counterparts.

Supplemental Tables E-3A, E-3B present clinical characteristics, demographics and pathological diagnoses of patients with IPAH and normal (rejected) donor controls (CTL) for lung tissue samples analyzed in this study (**Table E-3A** – for frozen OCT-embedded specimens and **Table E-3B** – for formalin-fixed specimens).

Suppl. Fig. E-8A (immunofluorescernt (IF) staining of frozen O.C.T-embedded sections) and **Fig.E-8B** (immunohistochemical (IHC) staining of formalin-fixed paraffin-embedded (FFPE) sections) demonstrates deposition of C3d (final degradation fragment of complement C3), which is most prominently observed in a perivascular-specific manner in the lungs of IPAH patients, but is minimal-to-none in the lungs of normal (rejected for lung transplant) donors (CO).

Supplemental Table E-4 lists proteins with prognostic relevance in idiopathic and hereditary PAH that were used to develop the *complement-PAH network*.

Suppl. Fig. E-9 demonstrates immunohistochemical staining for IgG in a medium sized pulmonary artery. The histochemical signal is present predominantly in the outer layers of the remodeled pulmonary artery (intima thickening highlighted by **arrows**). IgG deposition was also present in small arteries at the alveolar wall level (**arrowheads**). B. The deposition pattern of IgG is highlighted in this pulmonary artery with intima and media remodeling (**arrows**), with accentuation of deposition in the perivascular region (**arrowheads**), coinciding with that seen with C3d seen in **Figs. 9B-a** and **E-8B**.

Supplemental Table E-5 displays list of mouse and human TaqMan probes and primers used in the study.

REFERENCES:

1. Thurman JM, Lenderink AM, Royer PA, Coleman KE, Zhou J, Lambris JD, Nemenoff RA, Quigg RJ, Holers VM. C3a is required for the production of CXC chemokines by tubular epithelial cells after renal ishemia/reperfusion. *J Immunol* 2007; 178: 1819-1828.

2. Frid MG, Brunetti JA, Burke DL, Carpenter TC, Davie NJ, Reeves JT, Roedersheimer MT, van Rooijen N, Stenmark KR. Hypoxia-induced pulmonary vascular remodeling requires recruitment of circulating mesenchymal precursors of a monocyte/macrophage lineage. *Am J Pathol* 2006; 168: 659-669.

3. Jolles S, Orange JS, Gardulf A, Stein MR, Shapiro R, Borte M, Berger M. Current treatment options with immunoglobulin G for the individualization of care in patients with primary immunodeficiency disease. *Clin Exp Immunol* 2015; 179: 146-160.

4. Klein-Schneegans AS, Kuntz L, Fonteneau P, Loor F. Serum concentrations of IgM, IgG1, IgG2b, IgG3 and IgA in C57BL/6 mice and their congenics at the lpr (lymphoproliferation) locus. *J Autoimmun* 1989; 2: 869-875.

5. Thurman JM, Kulik L, Orth H, Wong M, Renner B, Sargsyan SA, Mitchell LM, Hourcade DE, Hannan JP, Kovacs JM, Coughlin B, Woodell AS, Pickering MC, Rohrer B, Holers VM. Detection of complement activation using monoclonal antibodies against C3d. *J Clin Invest* 2013; 123: 2218-2230.

6. Petrache I, Natarajan V, Zhen L, Medler TR, Richter AT, Cho C, Hubbard WC, Berdyshev EV, Tuder RM. Ceramide upregulation causes pulmonary cell apoptosis and emphysema-like disease in mice. *Nat Med* 2005; 11: 491-498.

7. Li M, Riddle SR, Frid MG, El Kasmi KC, McKinsey TA, Sokol RJ, Strassheim D, Meyrick B, Yeager ME, Flockton AR, McKeon BA, Lemon DD, Horn TR, Anwar A, Barajas C, Stenmark KR. Emergence of fibroblasts with a proinflammatory epigenetically altered phenotype in severe hypoxic pulmonary hypertension. *J Immunol* 2011; 187: 2711-2722.

8. Pugliese SC, Kumar S, Janssen WJ, Graham BB, Frid MG, Riddle SR, El Kasmi KC, Stenmark KR. A Time- and Compartment-Specific Activation of Lung Macrophages in Hypoxic Pulmonary Hypertension. *J Immunol* 2017; 198: 4802-4812.

Supplemental Fig. E-1

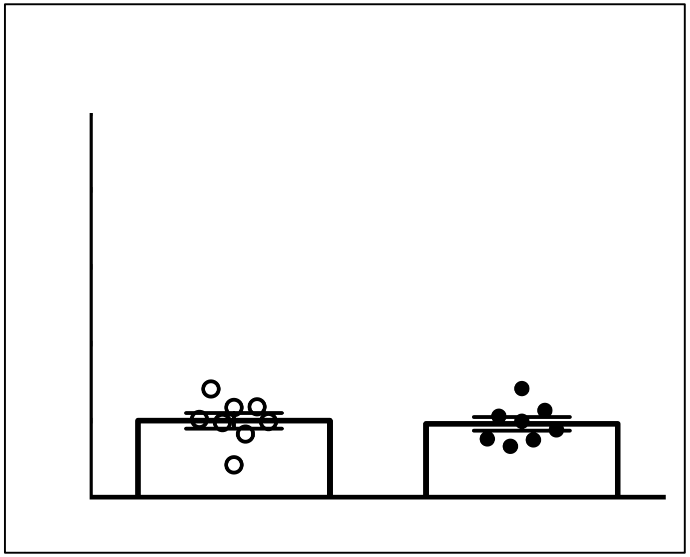

Supplemental Table E-1

IM-HX vs IM-SL

Gene	Common name	Gene detail	SL (FPKM)	4dHx (FPKM)	Fold change	P-value
ENSMUSG00000090231	**Cfb**	Complement factor B	13.03	41.53	3.19	0.0154
ENSMUSG00000026399	**Cd55/Daf**	Daf, decay accelerating factor for complement	16.48	6.68	-2.47	0.0141
ENSMUSG00000026365	**Cfh**	Complement factor H	100.37	70.03	-1.43	0.1417

Supplemental Fig. E-2

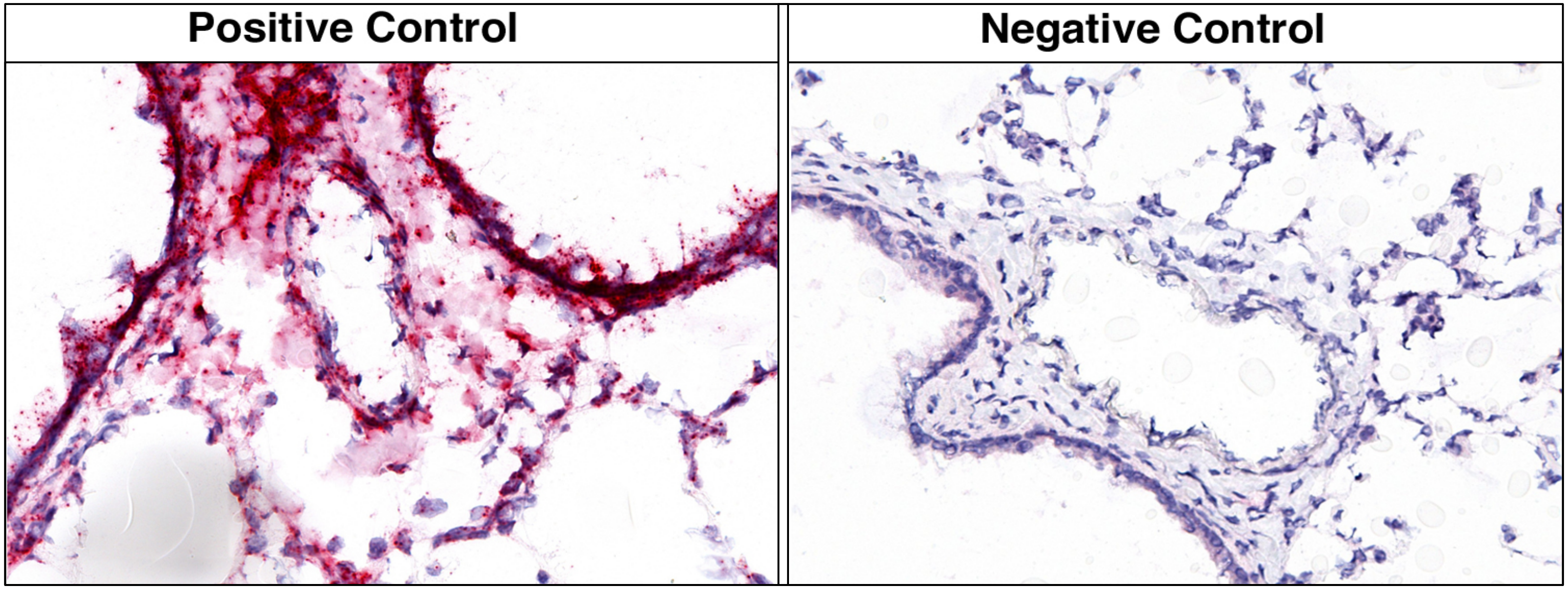

Supplemental Fig. E-3

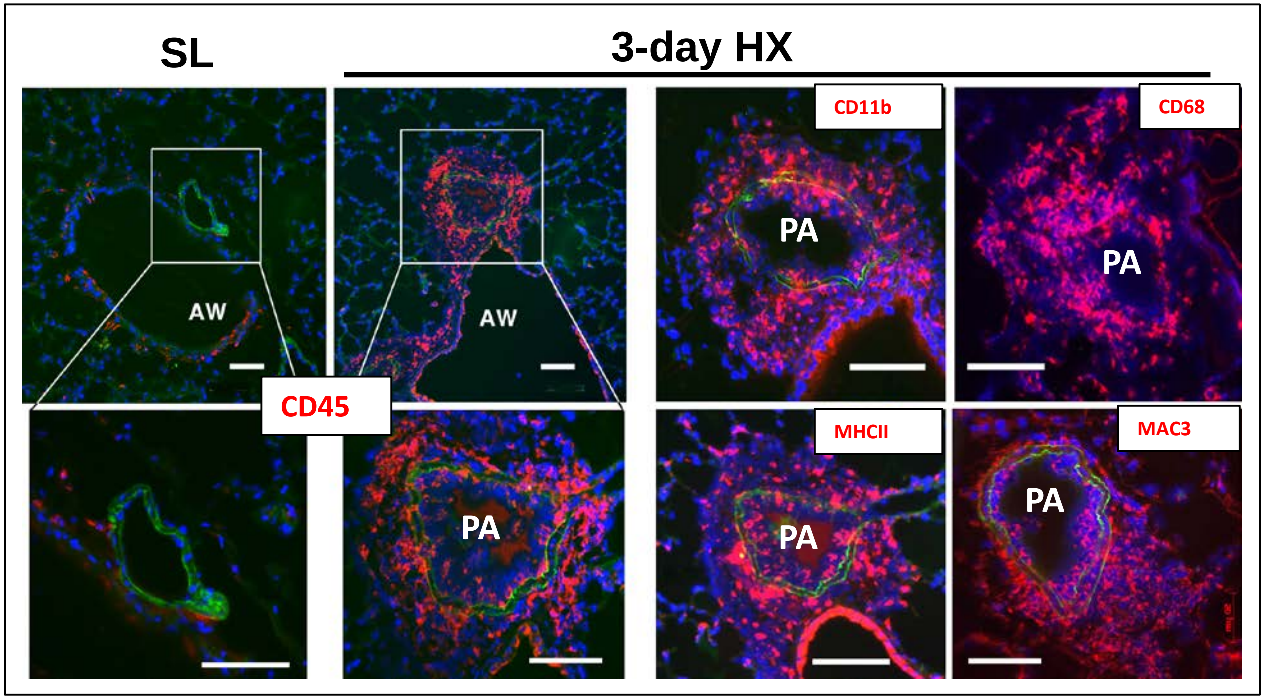

Supplemental Fig. E-4

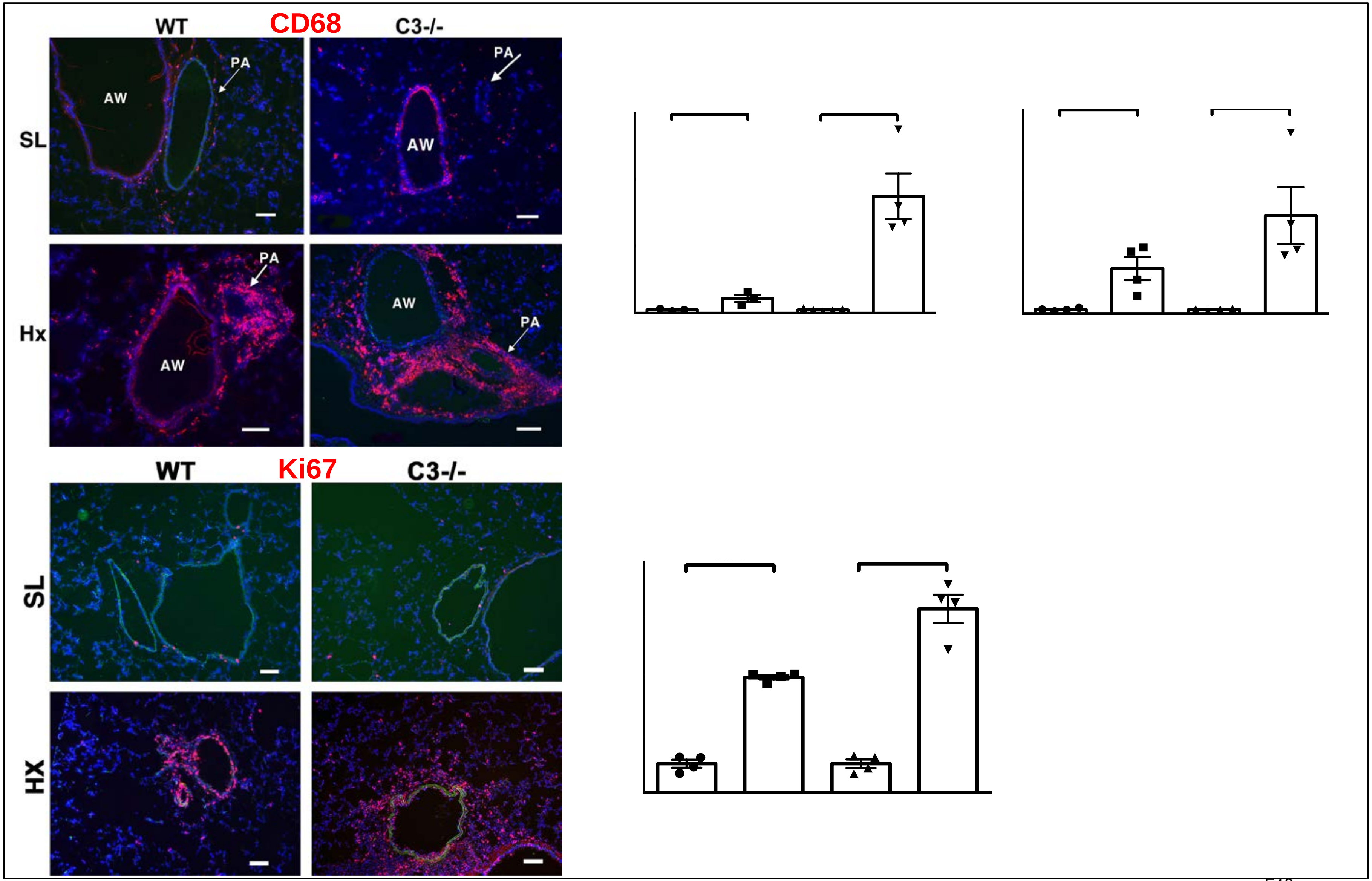

Supplemental Fig. E-5

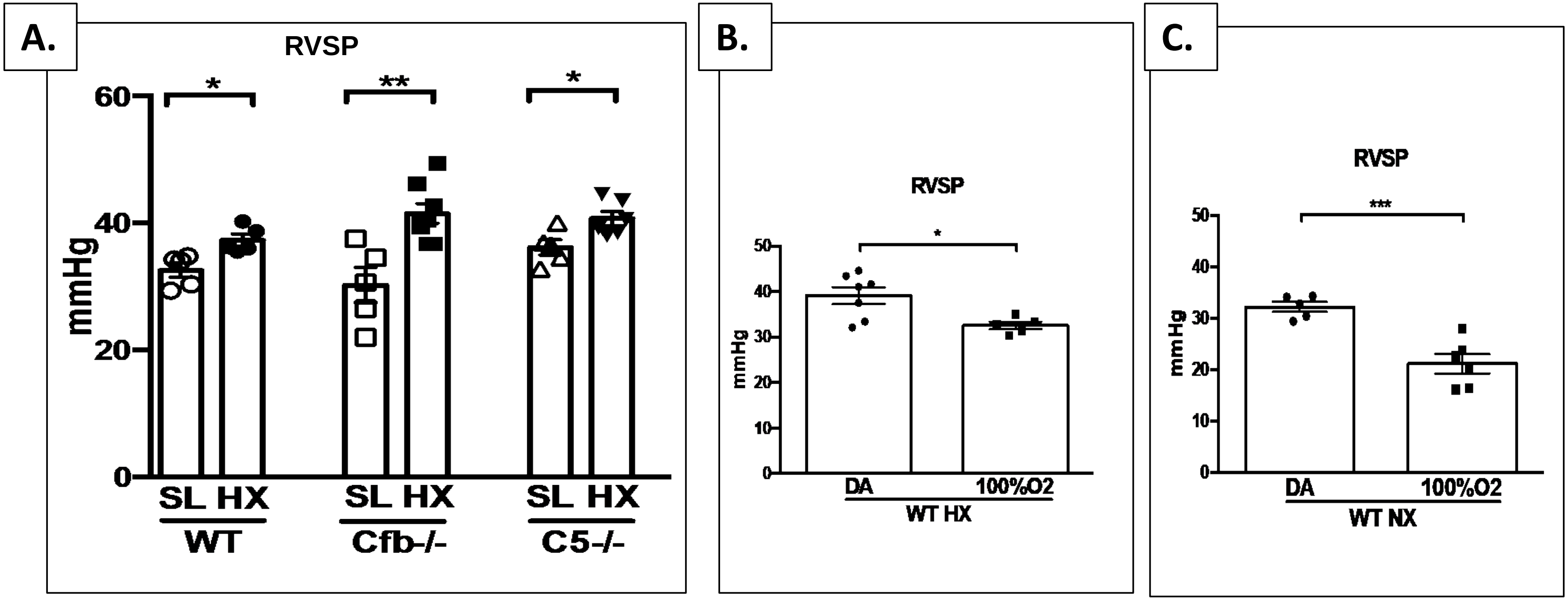

Supplemental Table E-2

Patient Diagnosis	Age	Gender	mPAP
IPAH	30.0	F	46
IPAH	39.0	F	55
IPAH	17.4	M	86
IPAH	35.0	F	65
IPAH	50.9	F	45

Supplemental Fig. E-6

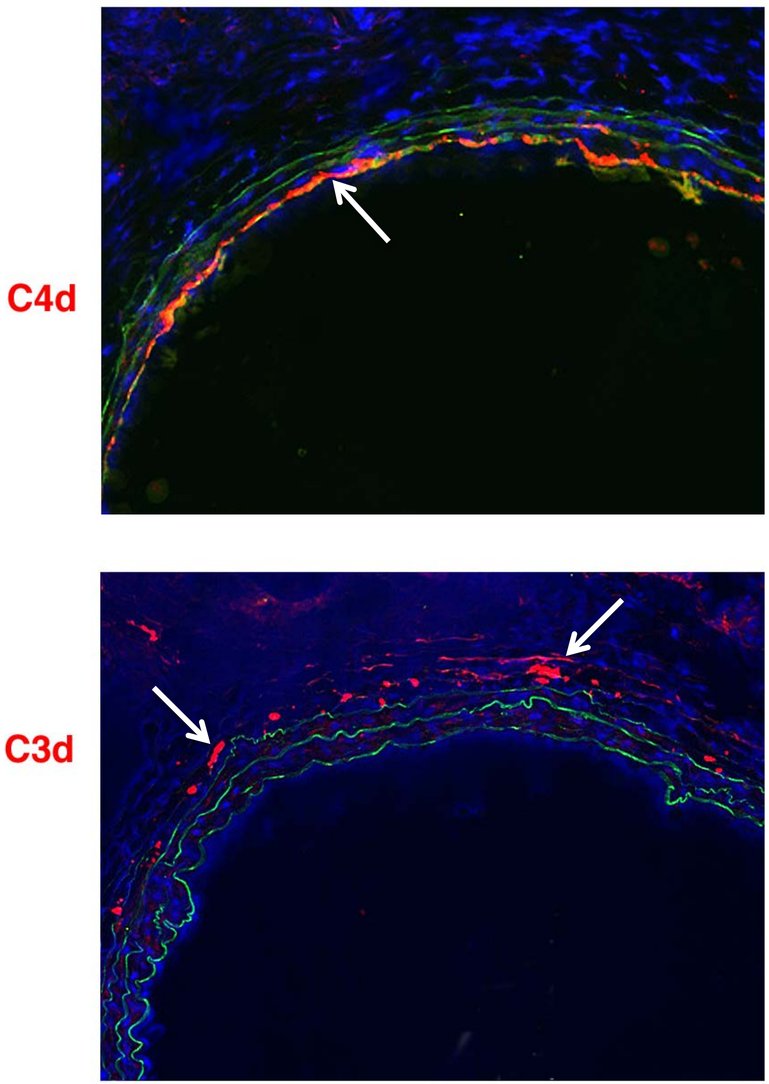

Supplemental Fig. E-7

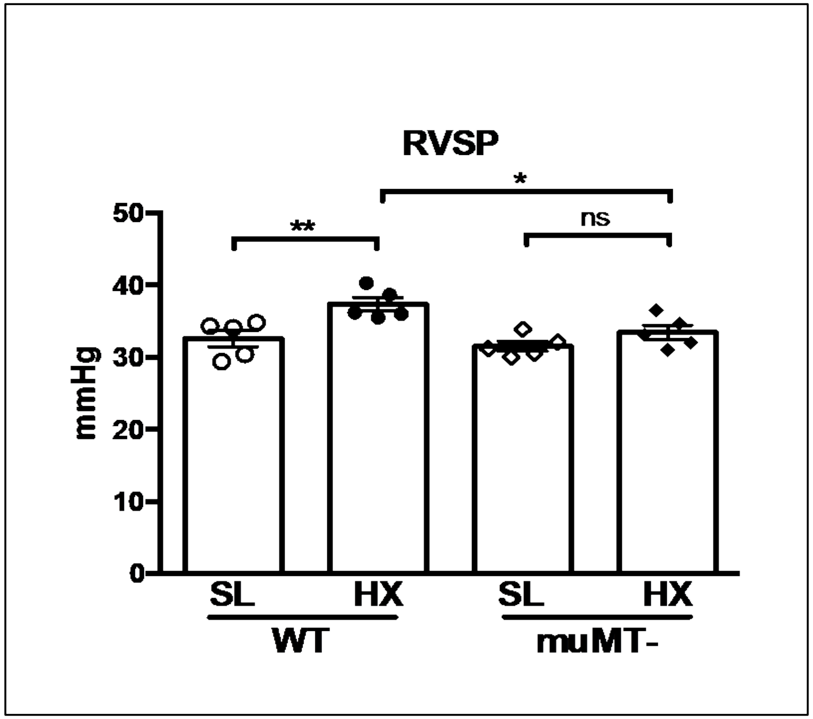

Supplemental Table E-3A

Patient Diagnosis	Age	Gender	Hemodynamics	
			mPAP	PVR
CTL	17.9	M	ND	ND
CTL	49.3	M	ND	ND
IPAH	10.83	F	62	17.5
IPAH	62.75	F	54	11.35
IPAH	56.4	F	51	6.78

Supplemental Table E-3B

Patient Diagnosis	Age	Gender	Hemodynamics	
			mPAP	PVR
CTL	54.5	M	ND	ND
CTL	57.0	F	ND	ND
CTL	56.0	F	ND	ND
CTL	41.4	F	ND	ND
CTL	49.3	M	ND	ND
IPAH	58.1	F	50	5.74
IPAH	14.0	M	76	29.78
IPAH	43.2	M	40	4.63
IPAH	36.5	F	55	13.33
IPAH	53.7	F	68	15.99
IPAH	56.9	F	57	11.41

Supplemental Fig. E-8A

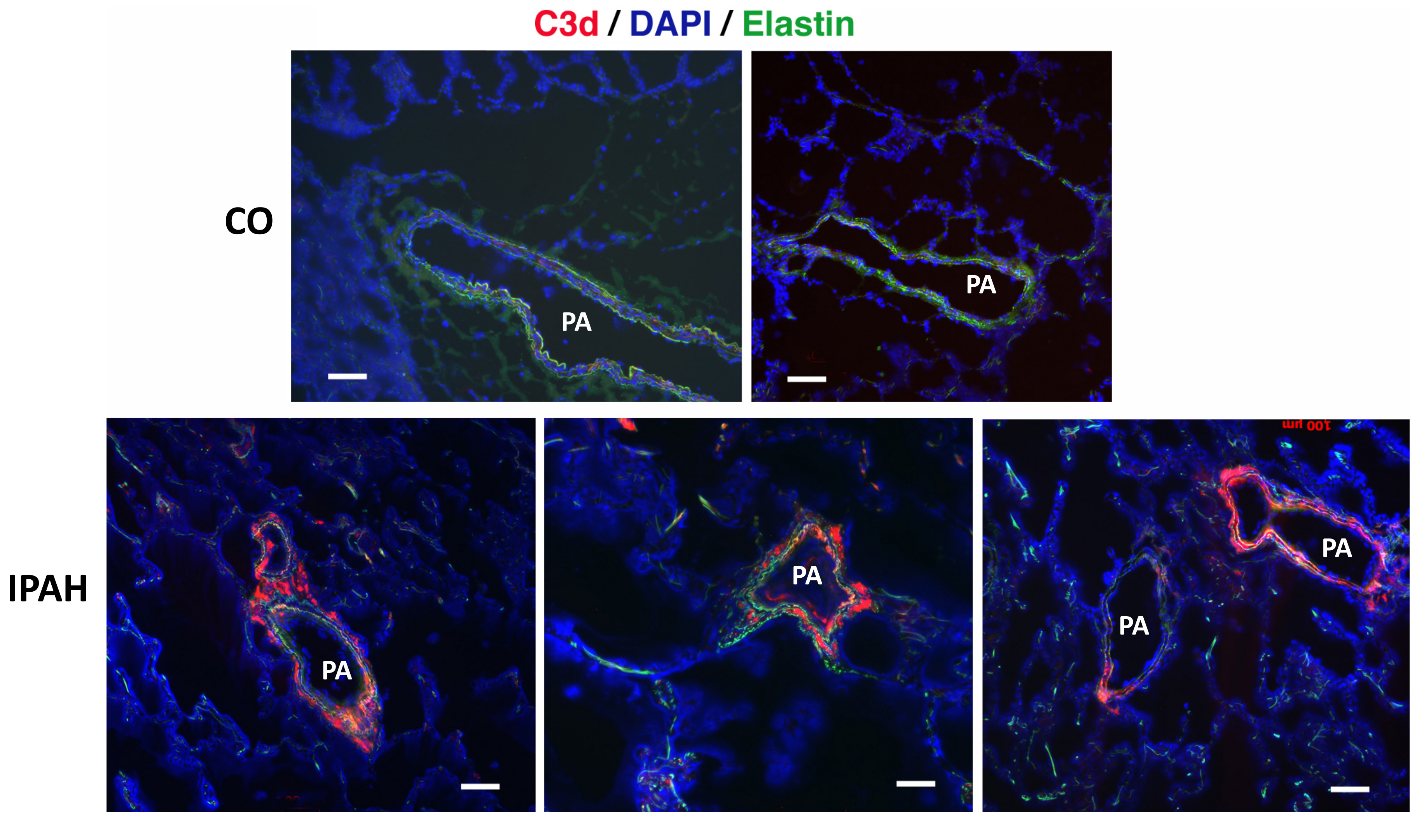

Supplemental Fig. E-8B

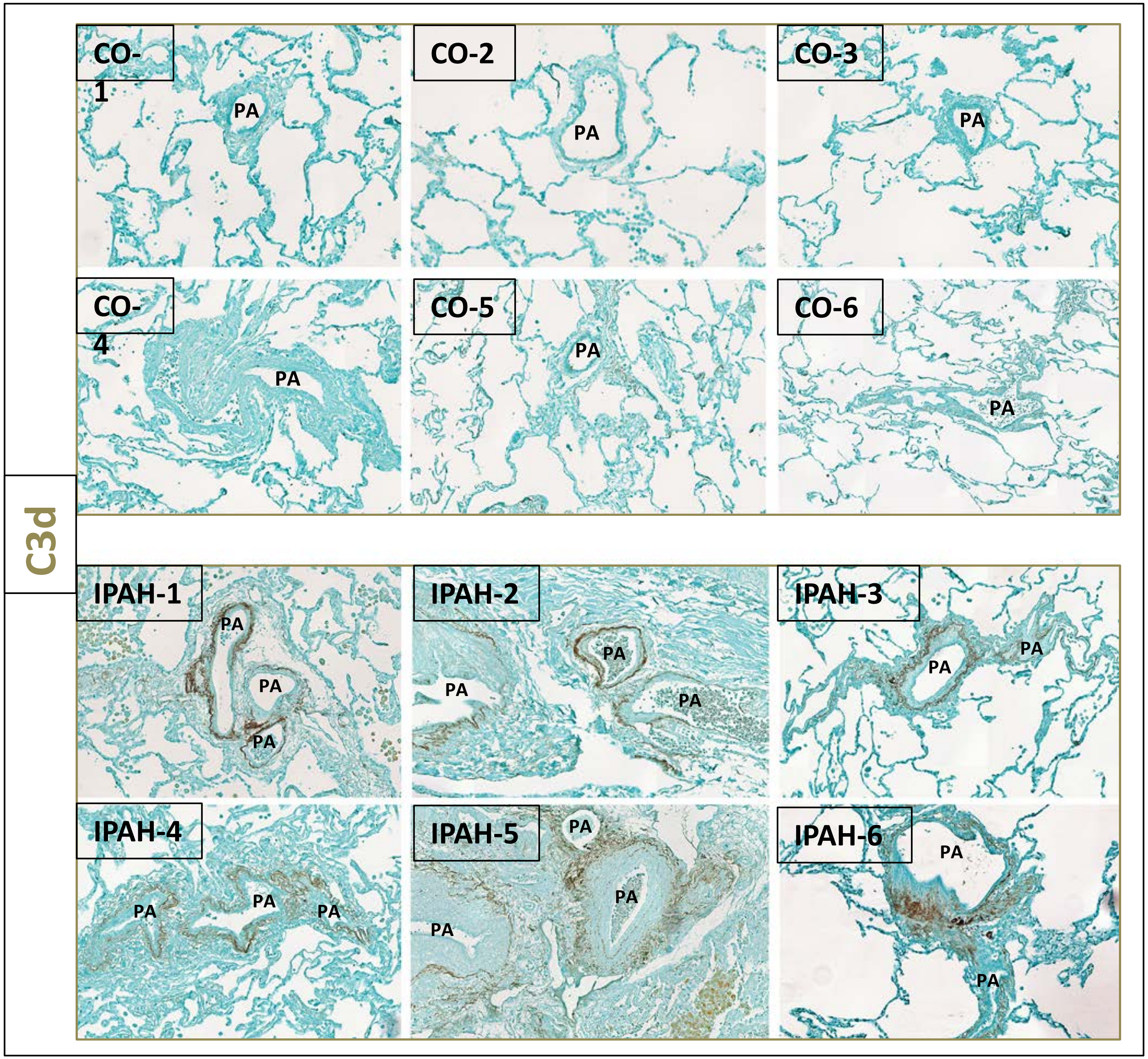

Supplemental Table E-4

Proteins	Gene ID
a1-Antitrypsin	SERPINA1
a2-Antiplasmin	SERPINF2
Afamin	AFM
Angiogenin	ANG
Angiopoietin-2	ANGPT2
Apo E	APOE
Apo E3	APOE
ASAH2	ASAH2
BMP-1	BMP1
BNP-32	NPPB
C3b	C3
C7	C7
CDON	CDON
CNDP1	CNDP1
Coagulation Factor V	F5
ENTP5	ENTPD5
Epo	EPO
Factor B	CFB
Factor D	CFD
Factor H	CFH
Growth hormone receptor	GHR
IGFBP-1	IGFBP1
IL-1	IL1A
IL-2 sRa	IL2RA
IL-22BP	IL22RA2
Kallikrein	KLKB1
Leptin	LEP
NRP1	NRP1
PARC	CCL18
PCI	SERPINA5
Plasminogen	PLG
Prekallikrein	KLKB1
Properdin	CFP
PTN	PTN
RET	RET
TFF3	TFF3
TIMP-1	TIMP1
TIMP-2	TIMP2
WKFN1	GPRASP2
XEDAR	EDA2R

Figure E-9.

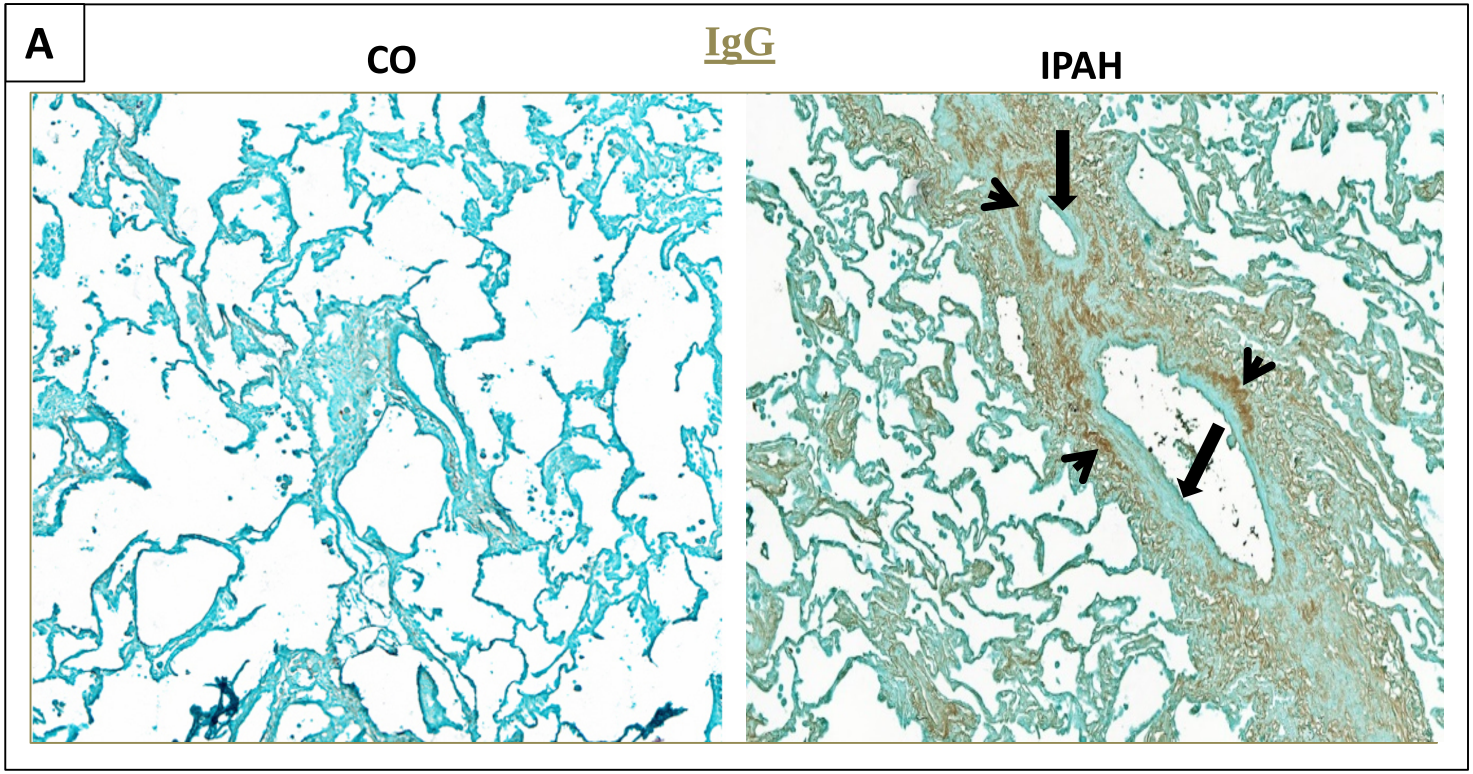

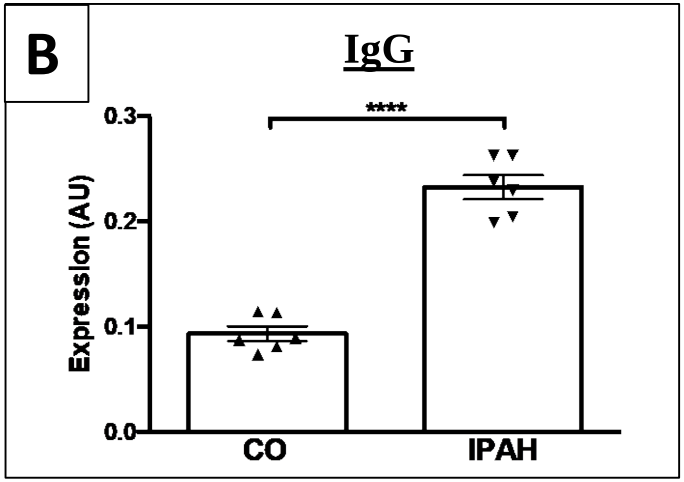

Supplemental Fig. E-9 (cont'd)

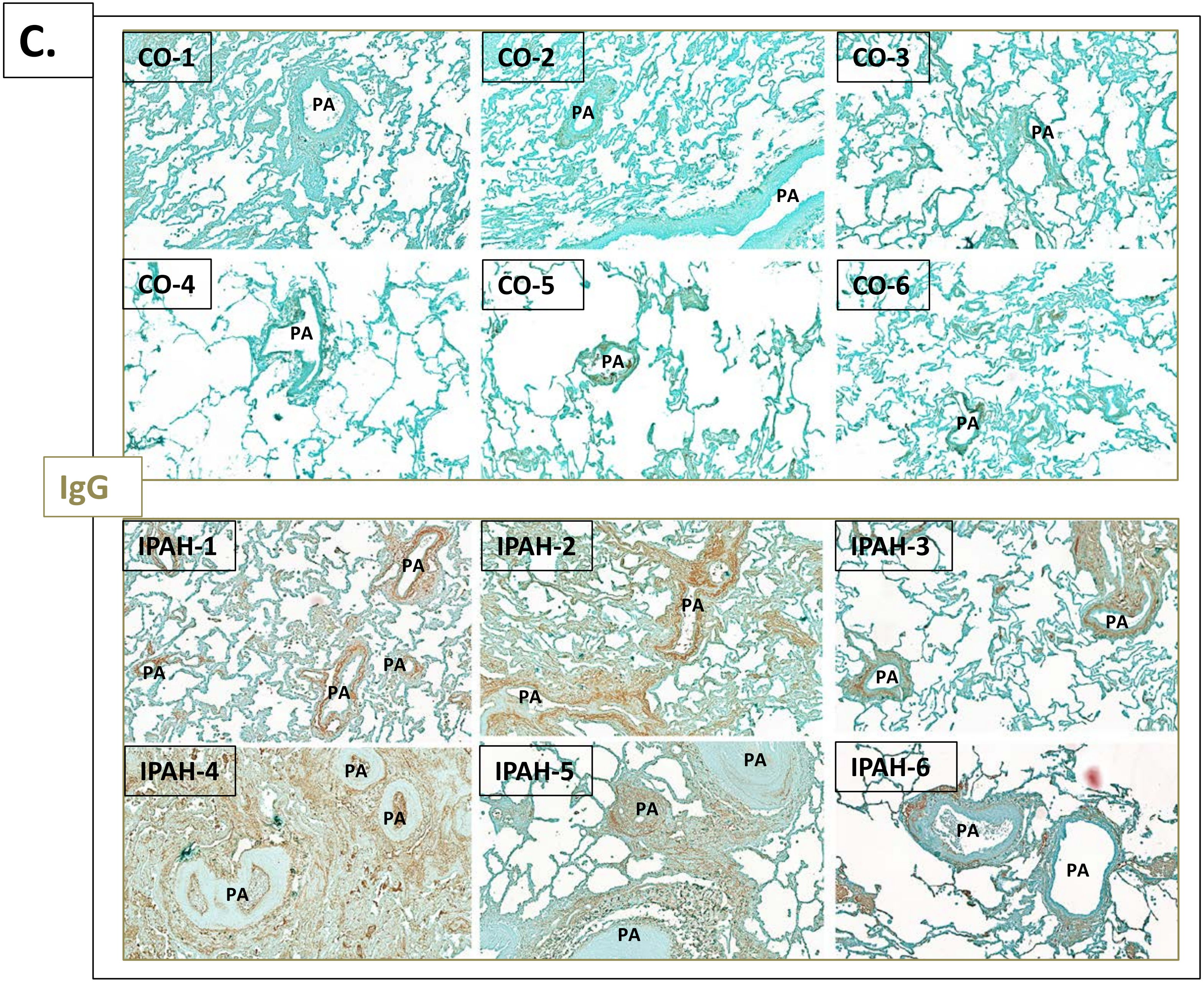

Supplemental Table E-5

Gene Name	Probe ID / Primer sequence
C3	Mm01232779_m1
C3ar1	Mm01184110_m1
C3ar1	Rn00583199_m1
C5ar1	Mm00500292_s1
C5ar1	Rn00586108_m1
Cfb	Mm00433909_m1
Cfh	Mm01299248_m1
Cd55/Daf	Mm00438377_m1
C4b	Primer-f; TGTCATCGAGTCTCCAGGAGG<br>Primer-r; AGGAGAAGGCAGATGATACAAAGC
Cd68	Mm03047343_m1
Csf2	Mm01290062_m1
CSF2	Hs00929873_m1
Ccl2	Mm00441242_m1
CCl2	Hs00234140_m1
Cdk1	Mm00772472_m1
CDK1	Hs00938777_m1
Hprt	Mm03024075_m1
HPRT	Hs02800695_m1
